# Altered ontogeny and transcriptomic signatures of tissue-resident pulmonary interstitial macrophages ameliorate allergic airway hyperresponsiveness

**DOI:** 10.3389/fimmu.2024.1371764

**Published:** 2024-06-25

**Authors:** Robert M. Tighe, Anastasiya Birukova, Yuryi Malakhau, Yoshihiko Kobayashi, Aaron T. Vose, Vidya Chandramohan, Jaime M. Cyphert-Daly, R. Ian Cumming, Helene Fradin Kirshner, Purushothama R. Tata, Jennifer L. Ingram, Michael D. Gunn, Loretta G. Que, Yen-Rei A. Yu

**Affiliations:** ^1^ Department of Medicine, Duke University, Durham, NC, United States; ^2^ Department of Cell Biology, Duke University, Durham, NC, United States; ^3^ Institute for Life and Medical Sciences, Kyoto University, Kyoto, Japan; ^4^ Department of Neurosurgery, Duke University, Durham, NC, United States; ^5^ Duke Center for Genomic and Computational Biology, Durham, NC, United States; ^6^ Department of Medicine, University of Colorado Anschutz School of Medicine, Aurora, CO, United States

**Keywords:** macrophages, asthma, ontogeny, single-cell RNA-seq, house dust mite

## Abstract

**Introduction:**

Environmental exposures and experimental manipulations can alter the ontogenetic composition of tissue-resident macrophages. However, the impact of these alterations on subsequent immune responses, particularly in allergic airway diseases, remains poorly understood. This study aims to elucidate the significance of modified macrophage ontogeny resulting from environmental exposures on allergic airway responses to house dust mite (HDM) allergen.

**Methods:**

We utilized embryonic lineage labeling to delineate the ontogenetic profile of tissue-resident macrophages at baseline and following the resolution of repeated lipopolysaccharide (LPS)-induced lung injury. We investigated differences in house dust mite (HDM)-induced allergy to assess the influence of macrophage ontogeny on allergic airway responses. Additionally, we employed single-cell RNA sequencing (scRNAseq) and immunofluorescent staining to characterize the pulmonary macrophage composition, associated pathways, and tissue localization.

**Results:**

Our findings demonstrate that the ontogeny of homeostatic alveolar and interstitial macrophages is altered after the resolution from repeated LPS-induced lung injury, leading to the replacement of embryonic-derived by bone marrow-derived macrophages. This shift in macrophage ontogeny is associated with reduced HDM-induced allergic airway responses. Through scRNAseq and immunofluorescent staining, we identified a distinct subset of resident-derived interstitial macrophages expressing genes associated with allergic airway diseases, localized adjacent to terminal bronchi, and diminished by prior LPS exposure.

**Discussion:**

These results suggest a pivotal role for pulmonary macrophage ontogeny in modulating allergic airway responses. Moreover, our findings highlight the implications of prior environmental exposures in shaping future immune responses and influencing the development of allergies. By elucidating the mechanisms underlying these phenomena, this study provides valuable insights into potential therapeutic targets for allergic airway diseases and avenues for further research into immune modulation and allergic disease prevention.

## Introduction

Macrophages are central mediators of immune responses due to their diverse functional repertoire in homeostasis, initiation, propagation, and resolution of inflammation. Macrophage functions depend on their location in tissue structures and cellular origin (1–7). In the lung, tissue-resident macrophages are present in unique tissue compartments (alveolar or interstitial) and have distinct ontogenies (embryonic or bone marrow-derived). Following lung injury, monocytes can be recruited to the lung and differentiate into macrophages (i.e., monocyte-derived macrophages), and their injury response is distinct from tissue-resident macrophages (8, 9). In addition, depending on the type and extent of lung injury, monocyte-derived macrophages can take up lung residence, replace tissue-resident macrophages, and, over time, acquire properties resembling tissue-resident macrophages (8, 10). Despite this observation, there is limited understanding of how lung injury-induced alteration in lung tissue-resident macrophage ontogeny impacts future lung immune responses. Since lung injury is widespread, understanding how these prior injury events alter future immune responses that lead to chronic lung disease is critical.

The present study investigated how lung injury alters the ontogeny of tissue-resident macrophages and its consequences for future immune responses. We established a model of repeated lipopolysaccharide (LPS)-induced lung injury, recovery to homeostasis, and then assessment of house dust mite (HDM)-induced allergic airway disease. We report that repeated exposure to LPS causes the replacement of embryonic-derived alveolar and interstitial macrophages with bone marrow-derived macrophages. After re-establishing homeostasis, we exposed these mice to house dust mite (HDM) and assessed allergic airway responses. We identified prior LPS exposure reduced allergic airway disease, including reduced airway hyperresponsiveness, mucus, Th2 cytokine production, bronchoalveolar lavage, and tissue eosinophilia. Using single-cell RNA sequencing, we observed LPS pre-exposure reduced CCR5 and IL8 signaling in alveolar macrophages regardless of ontogeny but suggestive of trained memory to LPS exposure. Additionally, we identified a subset of embryonic-derived interstitial macrophages expressing allergic genes primarily located in peribronchiolar lung regions diminished in LPS-pre-exposed mice. This suggests that altering pulmonary macrophage composition and phenotype by prior innate immune activation may confer protection against subsequent allergic airway disease.

## Materials and methods

### Experimental animals

C57BL/6, B6.129P2(C)-*Cx3cr1^tm2.1(cre/ERT2)Jung^*/J (Cx3cr1^CreER^) and B6.Cg-*Gt(ROSA)26Sor^tm14(CAG-tdTomato)Hze^*/J (tdTomato) were purchased from the Jackson Laboratory. Male Cx3cr1^CreER^ were crossed with tdTomato mice to generate a Cx3cr1^CreER^/tdTomato lineage reporter. To induce the lineage labeling during the embryonic stage, pregnant females were treated by oral gavage with either 2 mg/mL tamoxifen (Sigma-Aldrich) plus 0.7mg/mL progesterone (Sigma-Aldrich); 3 mg/mL tamoxifen plus 1.4 mg/mL progesterone; or 4 mg/mL tamoxifen plus 2.1 mg/mL progesterone at specified embryonic stage (E7.5 *vs.* E13.5). Due to the impact of tamoxifen on the pregnancy, progesterone was added to aid development and prevent spontaneous abortion. In addition, caesarian section and fostering were performed to allow growth to adulthood. As previously described, bone marrow chimeras were generated using busulfan (11). Experiments were conducted per National Institute of Health guidelines and protocols approved by the Animal Care and Use Committee at Duke University.

### LPS exposure, macrophage depletion, and allergic airway disease models

LPS (12.5 µg in 50 µL of PBS, Sigma-Aldrich) or PBS was administered by intranasal instillation (i.n.) under light isoflurane anesthesia. This occurred every other week for 3 doses. Following LPS or PBS exposure, the mice were allowed to recover for >8 weeks before acute HDM exposure. For the acute model, mice were challenged with 50µl of Dermatophagoides Pteronyssinus HDM (100µg total protein, Greer Laboratories Lot#296771, Lenoir, NC) or PBS intranasally (i.n.) on day 1, 7, and 14 under light isoflurane anesthesia. HDM Lot#296771 contains Der P1 113.21 µg/vial and endotoxin 28,750 EU/vial. Airway physiology measurements, bronchoalveolar lavage, and tissue harvests were conducted 48 hours after the last challenge.

### Airway physiology measurements

Airway responsiveness to acetyl-β-methacholine (MCh; Sigma-Aldrich) was measured 48h following the final HDM exposure using a computer-controlled small animal ventilator (FlexiVent FX, Scireq, Montreal, Canada) as we have previously described (12). Mice were anesthetized with urethane (1–2 g/kg; Sigma-Aldrich), tracheotomized and intubated with a metal endotracheal tube (18G blunt needle), placed on a 37°C water-heated pad, and mechanically ventilated at a rate of 150 breaths/min with a tidal volume of 10 ml/kg and a positive end-expiratory pressure (PEEP) of 3 cm H_2_O. To block spontaneous respiratory effort, mice were given pancuronium bromide i.p. (0.8 mg/kg; Sigma-Aldrich) five minutes before assessing airway responses. Both 1-second single-frequency and 3-second multi-frequency forced oscillation waveforms were applied using the Flexiware 7.6 software default mouse inhaled dose-response script to measure respiratory mechanics. The resulting pressure, volume, and flow signals were used to determine total respiratory system resistance (R_rs_) and elastance (E_rs_) or Newtonian resistance (Rn), tissue damping (G), and tissue elastance (H) as previously described (13, 14). MCh (10–100 mg/ml) was aerosolized using the FlexiVent FX aeroneb nebulizer attachment. The peak response at each dose was averaged and graphed along with each group’s average peak baseline measurement.

### Bronchoalveolar lavage fluid and analysis

As previously described, the BALF was obtained using the gravity fill method (12). The lungs are inflated to 20 cm H20, approximating total lung capacity with PBS+100µM EDTA+40 µM DTPA via gravity inflation three times, and the fluid is withdrawn with a syringe inserted into a side port of the tracheal tubing. BALF was spun to remove cells. The mean BALF volume was 2.936 mL (standard deviation +/- 0.1984). The BALF was concentrated to 0.25mL using a centrifugal filter (Millipore), and cytokines were measured using a custom Procartaplex (Invitrogen). Cell pellets were treated with RBC lysis buffer (BioLegend) and re-suspended in 1mL HyClone (Thermo Scientific). Total cells were counted with a K2 Cellometer (Nexcelom Bioscience) using AO/PI staining to exclude dead cells/debris. After staining with Hema 3 solution (Protocol, Kalamazoo, MI), counts of different immune cells were obtained under light microscopy. The cell count is normalized to the initial volume of BALF (cells/ml). Cell counts, differentials and cytokines were assessed by technician blinded to the exposure conditions.

### Flow cytometry

Flow cytometry was performed on the whole lung tissue using our previously published protocols (15, 16). In brief, mice were administered 50uL of 1000/mL heparin subcutaneously before euthanasia. Ten minutes after the injection, the mice were euthanized with isoflurane. The chest wall was opened, and the trachea cannulated. Following cardiac perfusion with 10mL of PBS to remove intravascular red cells, the lung was insufflated past total lung capacity with a digestion solution (1.5mg/ml of Collagenase A (Roche) and 0.4mg/ml DNase I (Roche) in HBSS plus 5% fetal bovine serum and 10mM HEPES). The lung tissue was excised and incubated at 37°C for 30 minutes with intermittent vortexing to generate a cell suspension. After digestion, the samples were washed and strained through a 70uM cell strainer and underwent red cell lysis (ACK RBC lysis solution). The cells were then manually counted and underwent staining for flow cytometry using a previously described flow cytometry panel and gating strategy [**Supplementary Table 1**, **Supplementary Figure S1**, and (15)] of established markers of lung immune cells (17). Flow cytometry was performed on a BD LSRII using BD FACSDiva software and then analyzed using FlowJo version 10 software.

### Histological analysis

Left lung lobes were isolated and fixed by gravity inflation (20 cm H_2_O) and immersion in 10% formalin. Lungs were paraffin-embedded, cut into 5µm sections, and stained by AML laboratories (Saint Augustine, FL). After individual staining, 10 images of randomly chosen variable-size airways were photographed at 10–20x magnification and analyzed in a blinded examination. Lung tissue inflammation was semi-quantitatively determined from hematoxylin and eosin (H&E) and Alcian blue-Periodic acid-Schiff (AB-PAS) stained sections using a four-tiered scoring system as previously described (18, 19). In addition, peri-bronchial fibrosis was determined from Masson’s trichrome staining using Image J (NIH) to calculate the percentage of peri-bronchial trichrome staining within the total tissue area containing the airway, including the airway’s lumen. Ten airways per animal were analyzed.

### Immunofluorescence

Representative unstained formalin-fixed paraffin-embedded (FFPE) sections (5 μm thickness) were stained using automated immunofluorescent techniques on BOND™ RXm Processing Module, utilizing the Bond Research Detection kit (Leica Microsystems) and Opal fluorophores (Akoya Biosciences) according to manufacturer instructions. In brief, FFPE sections were deparaffinized, hydrated with alcohol, and subjected to heat-induced epitope retrieval with Bond ER1 program. Slides were washed with Bond wash solution, and non-specific protein binding was blocked with protein block (5 min). For CD206 and Cathepsin K (CTSK) co-staining, the sections were sequentially incubated with CD206 (E6T5)(0.04ug/ml; Cell Signaling Technology) (30 min), Rabbit Polymer (10 min), Opal 520 (10 min) and CTSK (Rabbit anti-mouse CTSK polyclonal antibody(1:100; Novus Biological) (30 min), Rabbit Polymer (10 min), Opal 570 (10 min) Ab fluorophore combination. The nuclei were stained with spectral DAPI (4,6-diamidino-2-phenylindole) solution (Akoya Biosciences). Finally, the sections were cover-slipped using Vectashield HardSet Antifade mounting media (Vector Laboratories). Images were acquired using a Zeiss Upright 780 confocal microscope and processed using FUJI/Image J software.

### Microarray analyses

Yolk sac macrophages (CD45^+^CD11b^+^F4/80^+^) were sorted from the yolk sac layer of E9.5 embryos. AMØs (CD45^+^CD64^+^SSC^hi^CD11b^-^CD11c^+^SiglecF^+^), IMØs (CD45^+^CD64^+^SSC^hi^CD11b^+^CD11c^-^SiglecF^-^), Ly6C^+^ classical monocytes (CD45^+^CD64^lo^SSC^lo^CD11b^+^CD11c^-^Ly6C^+^), or Ly6C- non-classical monocytes (CD45^+^CD64^lo^SSC^lo^CD11b^+^CD11c^lo^Ly6C^-^) were sorted from lungs of mice. 10,000 cells were lysed using SuperAmp™ lysis buffer according to manufacturer instructions. Agilent Whole Mouse Genome Oligo Microarrays 8x60K were used to analyze transcriptomic profiles of sorted cells. Microarray processing, including RNA extraction, cDNA synthesis and labeling, hybridization, imaging, and data analysis with normalization, were performed by Miltenyi Biotec. Subsequent hierarchical clustering was performed using Partek bioinformatics software.

### Single cell RNA-sequencing

#### Mapping of reads to transcripts and cells

Lung cells from adult chimeric (CD45.1 *vs.* CD45.2), E13.5 lineage labeled adult Cx3cr1^CreER^/tdTomato mice were sorted into two fractions: Ly6G^-^B220^-^CD45.2^+^ vs. Ly6G^-^B220^-^CD45.1^+^. The presence of tdTomato in CD45.2 fractions was confirmed. Sorted cells from three animals (n=3) were pooled for library preparation and sequencing for each treatment condition. Libraries of cDNA from sorted cells were prepared by the Drop-seq platform as previously described by McCarroll Lab (http://mccarrolllab.com/dropseq) and sequenced on the Illumina HiSeq 4000 platform. Sequenced reads were mapped to the GRCm38v73 version of the mouse genome and transcriptome (20), binned, and collapsed onto the cell barcodes corresponding to individual cells using the Drop-seq pipeline v2.3.0 (21).

Raw digital expression matrices were generated separately for each of the eight samples and then merged into a single matrix. The Q*uickPerCellQC* function from the R package *scater* (22) v1.18.0 was used to define thresholds for identifying low-quality cells based on QC metrics (UMI count, number of genes detected, percentage of UMIs mapping to the mitochondrial genome). Cells containing >200 genes or <14% mitochondrial UMI were retained for downstream analyses. This filtered data set includes 32,919 cells expressing a total of 22,523 genes. The number of cells per sample ranged from 2,535 (PBS_PBS_CD45.2) to 6,159 (LPS_HDM_CD45.2), with a median of 4,124 cells. We used the R package *Seurat* v4.0.0 and its standard workflow for the integration of multiple samples to integrate the eight samples into a unique dataset (23) before clustering and visualization. Before integration, gene expression was normalized for each sample by scaling the total number of transcripts, multiplying by 10,000, and then log transforming (log-normalization). We then identified the 2,000 genes that were most variable across each sample, controlling for the relationship between mean expression and variance. Next, anchor genes were identified using the *FindIntegrationAnchors* function; then, *the IntegrateData function* was used to integrate the eight samples. Integrated data were scaled before performing principal component analyses (PCAs). To distinguish principal components (PCs) for further analyses, the JackStraw method was used to identify 30 statistically significant PCs. Subsequently, a shared nearest neighbor (SNN) modularity optimization-based clustering algorithm was used to determine cell clusters. This was implemented using the *FindNeighbors* function with 30 PCs and the *FindClusters* function with the Louvain algorithm. A range of resolutions was evaluated using the R package *clustree* v0.4.3 to generate a clustering tree. We identified clusters in resolution 0.4 to be particularly stable for higher resolutions. Resolution 0.4 corresponds to 18 cell clusters. Cluster-defining differentially expressed genes (DEGs) were identified using the Seurat function *FindConservedMarkers* for each cluster on the normalized gene expression before sample integration. R package *SingleR* v1.4.0 (24) was used to annotate cell types based on correlation profiles with bulk RNA-seq from the Immunological Genome Project (ImmGen) database (25). A total of 26,517 macrophages were identified, including 2032 PBS_PBS CD45.1 (2032 cells); PBS_PBS CD45.2 (2212 cells); LPS_PBS CD45.1 (3095 cells); LPS_PBS CD45.2 (3177 cells); 2521 PBS_HDM CD45.1 (2521 cells); 3885 PBS_HDM CD45.2 (3885 cells); LPS_HDM CD45.1 (4378 cells), and LPS_HDM CD45.2 (5307cells).

#### Macrophage identification

To examine macrophages, sub-clustering of macrophages was performed. First, we extracted subsets corresponding to clusters identified as macrophages (26,679 cells). Previously normalized UMI counts were used to identify 2,000 outlier genes on the mean variability plot for each sample; then, the integration of cells originating from each of the eight samples was performed similarly to the whole data set. Thirty statistically significant PCs were identified. Sub-clustering was then performed on the integrated subsets (resolution = 0.6). A total of 16 macrophage subclusters were identified. Cluster-defining DEGs were again identified using the Seurat function *FindConservedMarkers*. In the macrophage subsets, the three smallest clusters (399, 192, and 21 cells) were identified as small sets of remaining B cells, neutrophils, and mast cells, respectively. As a result, they were excluded from subsequent analyses. To separate the filtered subsets of macrophages into AMØs and IMØs, cells were annotated using the R package *SingleR* with bulk RNA-seq profiles from lung macrophages from Gibbings, Thomas, Atif, McCubbrey, Desch, Danhorn, Leach, Bratton, Henson, Janssen and Jakubzick (26). Cell-type specific markers confirmed the identity of AMØs and IMØs.

#### Sub-clustering of AMØs and IMØs

The data subset corresponding to AMØs (13,195 cells) was reintegrated and subclustered as described above (resolution of 0.1). Five AMØ subclusters were identified. Differential expression markers characterizing each subcluster were identified using the Seurat function *FindConservedMarkers*.

For IMØs (12,872 cells), a sub-cluster represented an artifactual cell cluster based on the high mitochondrial expression. We, therefore, examined the mitochondrial expression in the IMØ subset for each sample (**Supplementary Figure S2**). Five samples display a bimodal distribution of mitochondrial expression that was not detectable at the whole data set level. Thus, the mitochondrial percent expression threshold was lowered to 7.5% for the IMØ subset to filter additional low-quality cells. Reintegration and sub-clustering on this filtered IMØ subset (10,263 cells) were then performed (resolution of 0.3). None of the resulting seven subclusters identified were technical artifacts.

#### Gene set enrichment and pathway analysis

To further characterize the DEA results in the context of biological pathways, the ‘Core Analysis’ included in Ingenuity Pathway Analysis software (IPA, Ingenuity System Inc, USA, http://www.ingenuity.com) was run on differentially expressed genes (DEGs) comparing to PBS_PBS macrophages for each analysis (p-value < 0.05 and absolute value of log fold-change > 0.2). For both ‘Canonical Pathways’ and ‘Diseases & Functions’ downstream analyses, the p-value is calculated by IPA using a right-tailed Fisher’s Exact Test. The activation z-score predicts whether the upstream regulator exists in an activated or inactivated state. It is an independently calculated statistical measure of the correlation between relationship direction and gene expression (Z-score> 2 or < -2 is considered significant). The z-score cannot be calculated for all pathways or gene sets: there might be insufficient evidence in the IPA Knowledge Base for confident activity predictions across datasets or having fewer than four analysis-ready molecules in the dataset associated with the pathway.

## Statistics

Data for animal exposure studies were analyzed using GraphPad Prism 7 software (San Diego, CA). Complex data sets were analyzed using one-way or two-way ANOVA with assessment for multiple comparisons. Data points over 2 standard deviations from the mean were excluded from the data set. The level of significance for all tests was set at *P* < 0.05.

## Data availability

Data has been submitted to Bioproject (BioProject ID: PRJNA1049857). Reviewer link: https://dataview.ncbi.nlm.nih.gov/object/PRJNA1049857?reviewer=b0qaf0lln37bmj86789uc01oj9.

## Results

### Embryonic lineage labeling defines the unique ontogeny of resident pulmonary alveolar and interstitial macrophages

**Figure 1:**
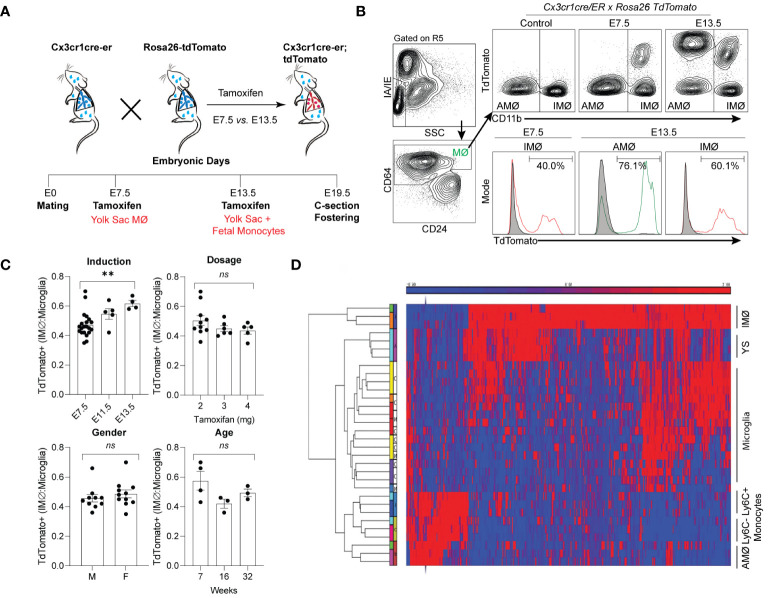
Time-mated lineage labeling of lung macrophages supports that interstitial macrophages (IMØ) are Yolk Sac-derived and persist into adulthood. **(A)** Overview of experimental design for time-mated lineage labeling in Cx3cr1cre-er;tdTomato mice induced with tamoxifen at E7.5 to label yolk sac progenitors or at E13.5 to label yolk sac and fetal monocytes. **(B)** Flow cytometry of whole lung tissue in 7-week-old mice following embryonic lineage labeling cells in the alveolar macrophage (AMØ) and IMØ compartments based on time of lineage labeling (E7.5 versus E13.5). Live, CD45^+^, Ly6G^-^, and CD64^+^ cells were then assessed for TdTomato expression versus CD11b to define AMØ (CD11b^-^) and IMØ (CD11b^+^). Histograms define representative percentages for TdTomato^+^ AMØ or IMØ in adult mice following induction. **(C)** Extent of lineage labeling in IMØ, defined as TdTomato^+^ cells in IMØ/microglia, was determined based on the time point of lineage induction, the tamoxifen dose, the sex of mice, and the time point of harvest after induction. **(D)** Microarray of sorted AMØ, IMØ, Ly6C^-^ and Ly6C^+^ monocytes, yolk sac (YS), and microglia reveals clustering of AMØ with monocytes and not IMØ that have greater overlap with YS. N=3–10 mice/group, experiments repeated >3 times, **p<0.005, ns=non-significant using ANOVA with post-hoc testing.

Tissue-resident lung macrophages are derived from embryonic lineages (i.e., primitive yolk sac macrophages or fetal liver monocytes recruited to the lung during embryonic development) or recruited from bone marrow-derived circulating monocytes (i.e., monocyte-derived macrophages) (27–29). Under homeostatic conditions, these tissue-resident macrophages are maintained through self-renewal for the animal’s lifespan or replaced by bone marrow-derived circulating monocytes (29–31). The degree and speed of embryonic-derived cell substitution by bone marrow-derived cells may depend on the organ and cellular location (29, 32–35). To address the ontogeny and maintenance of resident pulmonary macrophages, Cx3cr1^CreER^/tdTomato mice were treated with tamoxifen *in utero* to label primitive yolk sac progenies (E7.5) vs. both yolk sac- and fetal liver monocyte-derived progenies (E13.5). Lineage-labeled mice were allowed to age to adulthood to define the ontogeny of macrophages in adult mice (**Figure 1A**). With E7.5 labeling, approximately 40% of interstitial macrophages (IMØs, CD45^+^CD64^+^CD11b^+^SiglecF^-^) were tdTomato^+^ (**Figure 1B**), but 100% of the alveolar macrophage population (AMØ; CD45^+^CD64^+^CD11b^-^SiglecF^+^) were tdTomato^-^ (**Figure 1B**). At E13.5, 60% of IMØs and ~75–80% of AMØs were tdTomato^+^ (**Figure 1B**), suggesting that IMØs and AMØs have different ontogeny, and a substantial percentage of IMØs are derived from primitive yolk sac progenitors.

In most organs, yolk sac-derived tissue-resident macrophages are diluted or replaced by bone marrow-derived macrophages as the animals mature and age (36). To determine the persistence of yolk sac macrophage-derived IMØs and account for labeling efficiency, the percentage of E7.5 lineage-labeled tdTomato^+^ IMØs were normalized to lineage-labeled microglia, as microglia are derived from yolk sac macrophages and persist through the lifespan of animals (37). The ratio of lineage labeled IMØs to microglia remained relatively stable at all ages of animals examined (7, 16, and 32 weeks of age) (**Figure 1C**-Age). There were no alterations in the ratio based on the dosage of tamoxifen or the gender of the animal (**Figure 1C**-Dosage and Gender). A minimal increase in tdTomato^+^ IMØ proportion was observed based on tamoxifen-induction timing at E7.5 *vs.* E13.5 (**Figure 1C**-Induction), which might be due to improved labeling efficiency of IMØs at E7.5 or a minor contribution of the fetal liver monocyte precursors to the IMØ pool. The lineage labeled IMØs to microglia ratio remained relatively stable with different tamoxifen doses, animal gender, and age (**Figure 1C**-Dosage, Gender, and Age). These findings suggest that, under homeostatic conditions, a substantial portion of tissue-resident pulmonary IMØs, like microglia, are derived from yolk-sac progenitors and maintained through local self-renewal. To further confirm the ontogeny of resident pulmonary macrophages, the transcriptome of sorted AMØs, IMØs, microglia, yolk sac macrophages, Ly6C^+^ classical monocytes (Ly6C^+^ Mono), and Ly6C^-^ non-classical monocytes (Ly6C^-^ Mono) were examined by microarray analysis followed by hierarchical clustering (**Figure 1D**). The AMØs differed from primitive yolk sac progenitors and clustered with monocytes (Ly6C^+^ and Ly6C^-^). The IMØs were distinct from AMØs but shared transcriptomic signatures with primitive yolk sac progenitors. Conflicting reports exist regarding the ontogeny of embryonic-derived IMØs (yolk sac progenitors vs. fetal liver monocytes) (31, 38). However, our findings support that IMØs and AMØs have distinct ontogeny and genetic programming, where AMØs primarily derive from fetal liver monocytes, and a substantial population of IMØs derive from primitive yolk sac progenitors. Additionally, IMØs derived from embryonic progenitors persist through adulthood under homeostatic conditions.

### Altered pulmonary macrophage composition is associated with reduced allergic airway responses

**Figure 2:**
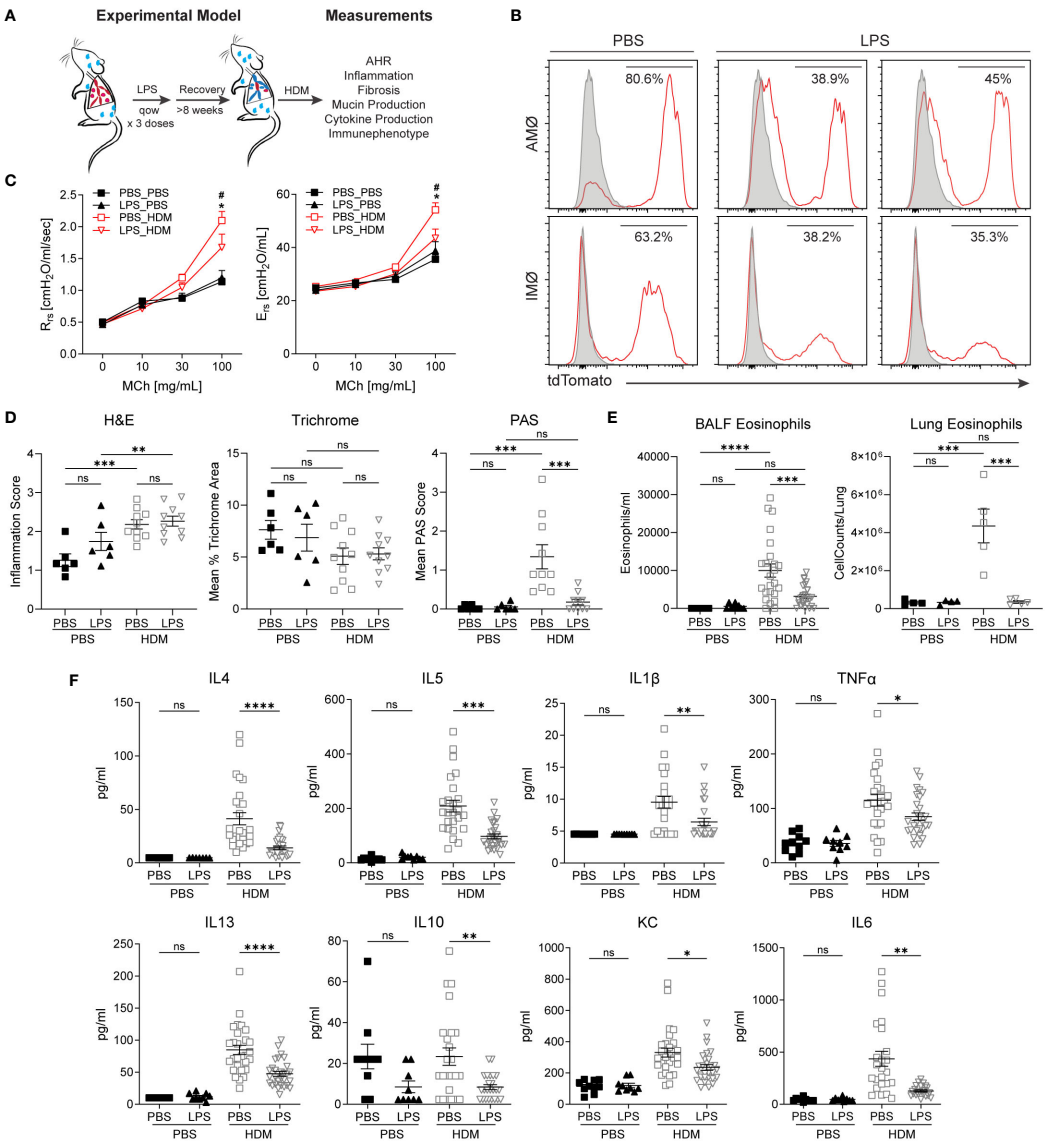
Replacement of embryonic-derived macrophages with bone marrow-derived macrophages following LPS exposure and recovery attenuates house dust mite (HDM) allergic airway responses. **(A)** Overview of experimental design to assess the impact of macrophage turnover on allergic airway responses. Mice underwent intranasal (i.n) LPS exposure (12.5µg) every other week for 3 doses. They were then allowed to recover for > 8 weeks. Following recovery, mice underwent acute HDM exposure and were assessed for phenotypic allergic airway responses. **(B)** Cx3cr1^CreER^/tdTomato mice underwent lineage reporter induction with tamoxifen at E13.5 and were allowed to age until 6 weeks. At 6 weeks of age, i.n. LPS or PBS exposure, as described above, was performed. Following >8 weeks of recovery from the exposure, lung tissues were harvested and processed for flow cytometry to define tdTomato expression in AMØ and IMØs. LPS causes the turnover of embryonic-derived macrophages to bone marrow-derived macrophages as determined by a reduction in the percentage of TdTomato+ cells. **(C-F)** LPS-mediated turnover reduces HDM-mediated allergic airway responses, including reduced airway hyperresponsiveness to increasing doses of methacholine (C, R_rs_-resistance, E_rs_-elastance), reduced periodic acid–Schiff (PAS) staining (a measure of mucus) without a difference in hematoxylin and eosin (H+E) or trichrome staining **(D)**, decreased lung tissue (as a % of CD45+ cells or total cells) and bronchoalveolar lavage fluid (BALF) eosinophils **(E)**, and decreased BALF cytokines including IL-4, IL-13, IL-1β, TNF-α, IL-5, IL-10, KC and IL-6 **(F)**. n=10 mice/group in the PBS/PBS and LPS/PBS groups and n=30 mice/group in the PBS/HDM and LPS/HDM groups. *p<0.05 when compared to PBS control or ^#^p<0.05 when compared between PBS-HDM and LPS-HDM groups by 2-way ANOVA with testing for multiple comparisons. *p<0.05, **p<0.005, ***p<0.0005, ****p<0.00005 for other comparisons by 1-way ANOVA or Students T-test, n.s., non-significant.

When the niche of tissue-resident macrophages is disturbed through experimental depletion or inflammation, bone marrow-derived macrophages adopt a tissue-resident phenotype with self-renewal capabilities (8, 32, 33, 39–41). After restoring homeostasis following environmental perturbation, bone marrow-derived macrophages often closely resemble those of unperturbed tissue-resident macrophages (8, 41). However, it remains uncertain whether altering the ontogenetic composition of tissue-resident macrophages affects the immune response to subsequent insults. To investigate this, we induced the replacement of embryonic-derived tissue-resident macrophages with bone marrow-derived ones. After homeostasis was re-established, we evaluated the effects of this switch from embryonic to bone marrow-derived macrophages on allergic airway responses to HDM (**Figure 2A**). Macrophage ontogenetic alteration was induced by intranasal exposure to LPS every other week for three doses. The mice were allowed to recover for > 8 weeks to ensure complete resolution of LPS-induced acute inflammation. To assess the efficacy and persistence of replacement at more than 8 weeks after LPS exposure, we examined the change in the proportion of tdTomato^+^ AMØs and IMØs in E13.5 embryonically labeled Cx3cr1CreER/tdTomato mice. Compared to PBS control mice, LPS-exposed mice showed approximately a 50% reduction in lineage-labeled tdTomato^+^ AMØs or IMØs, indicating the recruitment and persistence of bone marrow-derived macrophages (**Figure 2B**).

We assessed whether prior LPS exposure and associated macrophage alteration impacted allergic responses to the HDM challenge. While there were no significant differences in airway responses to methacholine challenges between PBS or LPS pre-exposed animals but not sensitized to HDM (*a.k.a.* PBS_PBS or LPS_PBS), we observed that mice pre-exposed to LPS and then HDM (LPS_HDM, red open triangle), when compared to mice exposed to PBS and then HDM (PBS_HDM, red open box), had reduced airway physiologic responsiveness, including resistance and elastance (**Figure 2C**). While LPS pre-exposure did not impact the degree of total inflammatory cell infiltrate (H&E) or peri-bronchial fibrosis (Mean % Trichrome), replacement by bone marrow-derived macrophages in the setting of repeated LPS exposure is associated with reduced mucus accumulation in response of HDM exposure (Mean PAS score) (**Figure 2D**; **Supplementary Figure S3**). Additionally, while LPS pre-exposure did not impact overall HDM-induced inflammatory cell infiltration assessed by BALF total cell, macrophage, neutrophil, lymphocyte counts, and proportion of immune cells in lung tissues, there was a significant reduction in eosinophil numbers and proportion in BALF and lung tissues in LPS_HDM animals (**Figure 2E**; **Supplementary Figures S4A, B**). Consistent with decreased eosinophils, there was a reduction in T helper 2 (Th2) cytokines in BALF, including IL-4, IL-5, and IL-13, which are known to modulate eosinophil trafficking and functions (**Figure 2F**). The absence of significant differences between PBS and LPS pre-exposed mice without HDM challenge by histologic scoring of inflammation (**Figure 2D**; **Supplementary Figure S3**), immune cell counts in bronchoalveolar lavage fluid (BALF) (**Figure 2E**, **Supplementary Figure S4A**), immune cell proportions and macrophage counts in lung tissues by flow cytometry (**Figure 2E**, **Supplementary Figures S4B, C**), and cytokine analyses of BALF (i.e., IL-1β, IL-4, IL-13, IL-1β, IL-5, IL-6, IL-10, IL-13, TNFα, and KC)(**Figure 2F**) are consistent with the absence of persistent inflammation resulting from prior LPS-exposure before subsequent HDM challenge. These data support that prior LPS exposure, which alters the ontogenetic composition of AMØs and IMØs, reduces HDM-induced mucin production, Th2 cytokines, and eosinophil infiltration, and improves allergic airway responses to HDM.

### Single-cell RNA sequencing reveals a unique lung resident IMØ subset exhibiting allergic transcriptomic signatures

**Figure 3:**
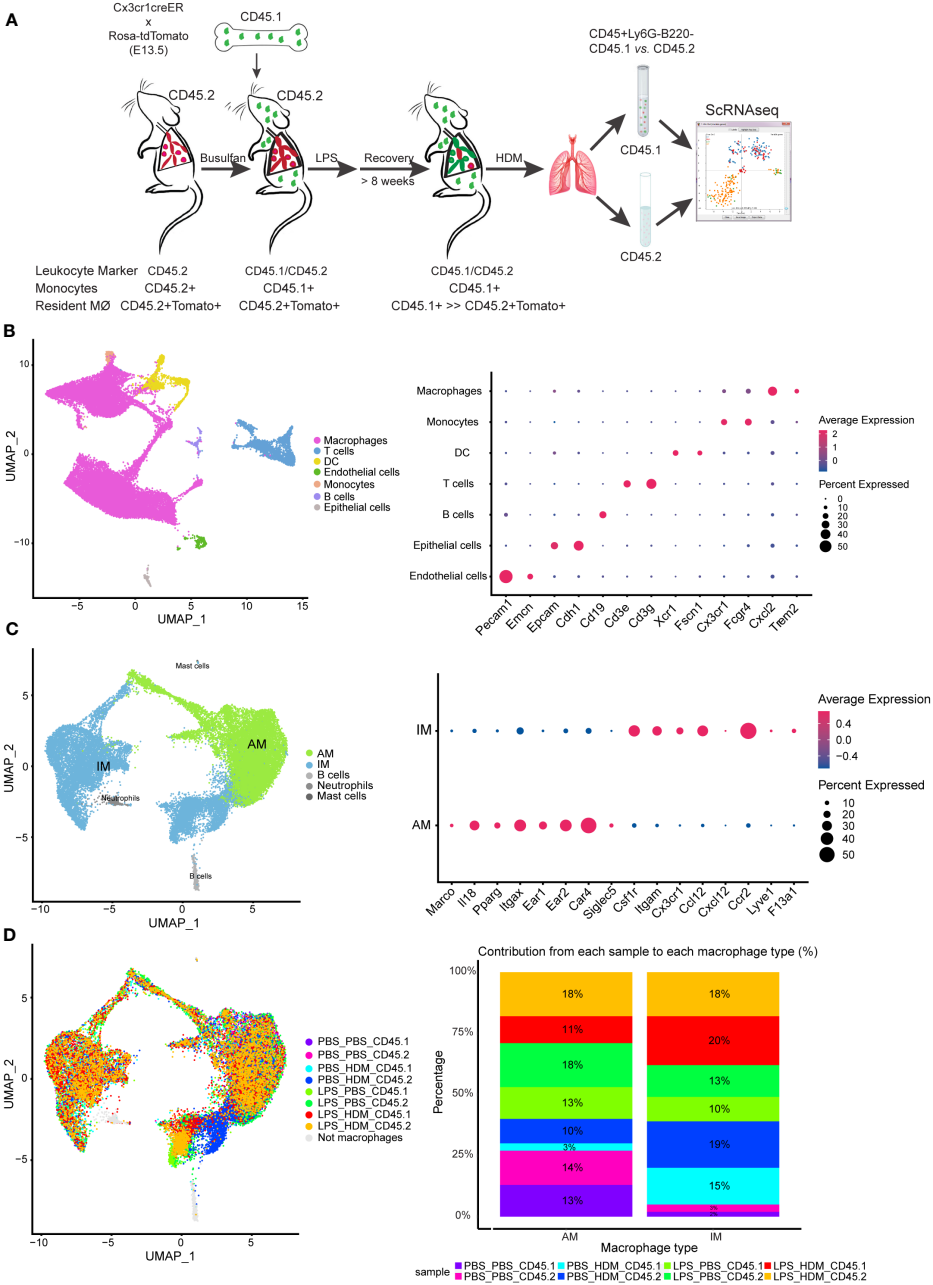
Single cell RNA sequencing of immune cells allows segregation of alveolar and interstitial macrophages and identifies unique clustering based on exposure conditions. **(A)** Bone marrow chimeras were generated from lineage labeled Cx3cr1^CreER^/tdTomato mice by injecting CD45.1 donor cells into CD45.2 recipients to define resident (CD45.2) and recruited (CD45.1) cell populations. Mice were exposed in the following groupings PBS_PBS, PBS_HDM, LPS_PBS, and LPS_HDM. Following exposures, lungs were harvested and processed for sorting. Live, Ly6G^-^, B220^-^ cells were segregated based on CD45.1 from CD45.2 expression and processed for single cell RNA-seq. **(B)** Clustering of immune cells based on defined markers identifies macrophages (pink), dendritic cells (DC, yellow), monocytes (orange), and T cells (blue). **(C)** Sub-clustering of macrophages segregates interstitial (IM, blue) from alveolar macrophages (AM, green) based on annotation using established markers. **(D)** Display of sub-clustering of macrophages based on exposure condition and whether the cells are recruited (CD45.1) or tissue-resident (CD45.2) identifies distinct clustering based on exposure and origin. The bar graph reveals that IMs increase as a percentage in response to exposures compared to the PBS_PBS or LPS_PBS groups. Following the flow sorting, data were pooled from individual sorted mouse lung samples (n=3 mice per exposure group).

To determine the impact of LPS pre-exposure and macrophage ontological alterations on HDM allergic responses, we performed single-cell RNA-sequencing (ScRNAseq) to define immune cell composition and gene expression. To define pulmonary macrophage ontogeny, we generated bone marrow chimera by transferring CD45.1 bone marrow donor cells into busulfan-treated CD45.2, embryonic-labeled (at E13.5) Cx3cr1^CreER^/tdTomato recipient mice (**Figure 3A**). We have previously shown that busulfan treatment induces efficient bone marrow myeloid ablation while having minimal effects on tissue-resident pulmonary macrophages (8, 11). Thus, we can clearly distinguish embryonic-derived resident macrophages (CD45.2^+^) from those arising from the bone marrow compartment (CD45.1^+^). After engraftment, consistent with preservation of tissue-resident pulmonary macrophages following busulfan treatment, >80% of AMØs and approximately 40% of IMØs were identified as CD45.2+ (host origin) (**Supplementary Figure S4D**). Chimeric mice were exposed to PBS or LPS every other week for 3 doses, allowed to recover for >8 weeks, and exposed to PBS or HDM. After LPS exposure, approximately 25% of AMØs and only 5–10% of IMØs were CD45.2+ after repeated LPS exposure consistent with altered ontogenetic composition following LPS exposure (**Supplementary Figure S4D**).

For ScRNAseq, myeloid cells were enriched, and embryonic- and bone marrow-derived cells were segregated by flow sorting for live, CD45^+^ Ly6G^-^ B220^-^ cells and then separated by CD45.1 vs. CD45.2 (**Figure 3A**). ScRNAseq of sorted cells identified distinct populations of cells based correlation profiles with bulk RNA-seq from the Immunological Genome Project (ImmGen) database (25) and confirm by cell type-associated markers. Clustering identified expected clusters of macrophages (*Trem2* and *Cxcl2*), T cells (*Cd3e* and *Cd3g*), dendritic cells (*Xcr1* and *Fscn1*), and monocytes (*Cx3cr1*) (**Figure 3B**). Small clusters of residual endothelial cells, B cells, and epithelial cells were observed which were excluded from subsequent analyses (**Figure 3B**). To define if there are unique clusters based on exposure condition and ontogeny, macrophages were subclustered into AMØs (*Itgax* and *Car4*) and IMØs (*Itgam*, *Csf1r*, *Cx3cr1*, *Ccr2*, and *Lyve1*) groupings (**Figure 3C**) as well as subclustered based on both exposure conditions (PBS *vs*. LPS, and PBS *vs.* HDM) and ontogeny (CD45.1 *vs.* CD45.2)(**Figure 3D**). Even though there were no significant pathway differences by IPA analyses (defined as Z score >2 or <2) between IMØs from the PBS or LPS pre-exposed animals without subsequent HDM challenge, following HDM exposure, we observed an expansion of IMØs in a specific cluster of cells (**Figure 3D**, dark blue) arising primarily from CD45.2 embryonic-derived interstitial macrophages in the setting of the HDM challenge. These observations suggest the unique macrophage clustering based on ontogeny and exposure conditions.

### Differential effects of ontogeny on alveolar and interstitial macrophages in allergic airway responses

**Figure 4:**
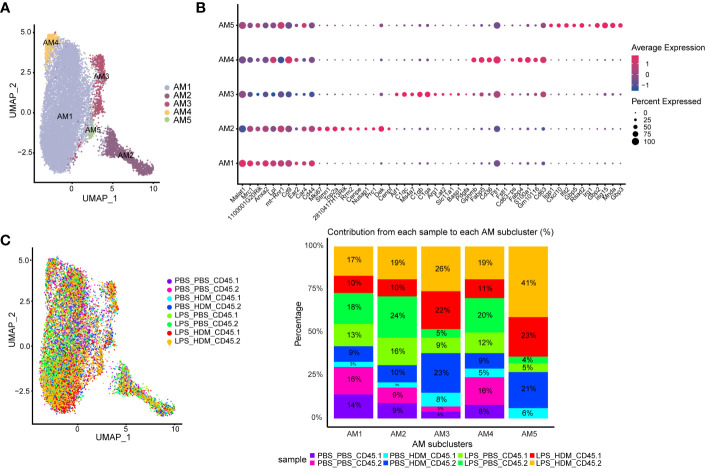
Alveolar macrophage sub-clustering reveals unique clusters with distinct gene expression patterns without evidence of distinct clustering based on ontogeny. **(A)** Alveolar macrophages (AM) are sub-clustered, identifying 5 unique clusters (AM1–5) defined by individual gene expression patterns **(B)**. **(C)** An overlay of the clusters based on exposure and CD45.1 (bone marrow-derived) or CD45.2 (tissue-resident) group in the UMAP plot, as well as a bar graph depicting or percentage contribution by each sample to individual AM subclusters. Data do not support clear cluster segregation based on ontogeny. Data were pooled from individual sorted mouse lung samples (n=3 mice per exposure group).

AMs and IMs have been implicated in regulating allergic asthma responses; thus, we performed further analyses of both AMØs and IMØs to determine how altering the ontogenetic milieu affects their transcriptomic profiles. Sub-cluster analysis of AMs revealed 5 unique clusters (AM1–5; **Figures 4A, B**). Based on differential gene expression and Ingenuity pathway (IPA) analysis (**Supplementary Figures S5**, **S6A**), AM1 consisted primarily of cells that down-regulated pro-inflammatory and proliferative processes (e.g., interferon signaling, chemokine signaling, neuroinflammatory signaling, mitosis, metaphase signaling, and cell cycle control of chromosome replication); AM2 represents a cluster of proliferating cells (e.g., Mki67; DNA replication, mitosis, and cell cycle progression); and AM3–5 participated in immune responses (*e.g.*, myeloid cell activation, chemotaxis/migration/accumulation, phagocytosis, or systemic autoimmune syndrome). Overlaying clusters based on exposure conditions and ontogeny did not reveal unique condition/ontogeny-specific clustering by UMAP or the individual cell/cluster percentages (**Figure 4C**). No prominent contribution of macrophage ontogeny (CD45.2 *vs.* CD45.1) was observed to HDM responses; however, DEG-based clustering revealed differences between the pre-HDM exposure conditions (PBS vs. LPS pre-exposure) (**Supplementary Figures S5A–E**). This data suggests that LPS pre-exposure, but not ontogeny, modulates AMØs phenotype in response to HDM challenge.

**Figure 5:**
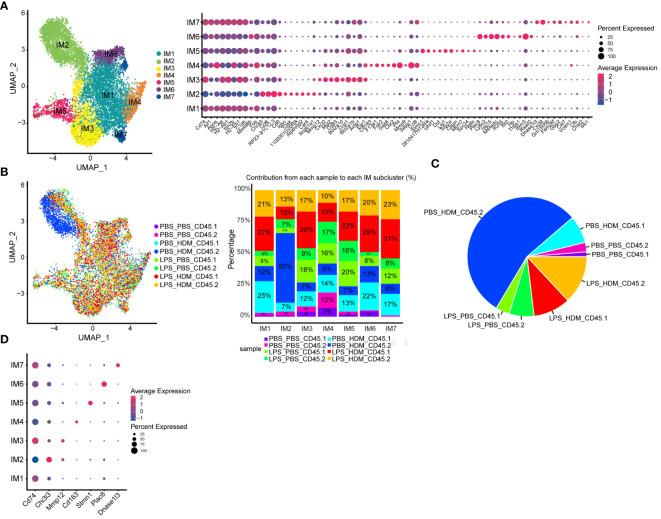
Interstitial macrophage sub-clustering reveals a unique IMØ subcluster that segregates by exposure condition and ontogeny and is defined by expression of Chi313. **(A)** Sub-clustering of interstitial macrophages (IM), defined by individual cellular markers, identifies seven unique clusters (IM1–7). **(B)** Overlay of IM clusters on UMAP and bar graph based on exposure condition and cell ontogeny identifies that IM2 cluster segregates based on CD45.2 (resident origins) and HDM exposure, **(C)** Pie chart demonstrating the distribution of the IM2 cluster based on the exposure condition and cell origins. This highlights that tissue-resident CD45.2 IMØ from the PBS_HDM exposed mice as the predominant constituent of the IM2 cluster. **(D)** Top gene expression for each cluster highlights that cluster IM2 is defined by gene expression for Chi313. Following the flow sorting, data were pooled from individual sorted mouse lung samples (n=3 mice per exposure group).

Analyses of IMØs identified 7 distinct clusters (**Figure 5A**). Similar to AMØ clusters, differential gene expression and IPA analyses define cells that down-regulated proliferative pathways (IM1), upregulated proliferation pathways (IM5), and cells involved in overlapping inflammatory pathways (IM3, 4, 6, and 7; e.g., iNOS, IL-1, IL-6, and Trem-1 pathways) (**Figure 5A**; **Supplementary Figure S6B**, and data not shown). Interestingly, IM2 consisted mainly of the population of previously observed cells (dark blue) that arise primarily from CD45.2 embryonic-derived Interstitial macrophages following the HDM challenge (CD45.2 PBS_HDM, blue in **Figures 3D**, **5B, C**). This response was not observed in animals re-challenged with PBS regardless of ontogeny or prior LPS exposure (CD45.1 PBS_PBS, CD45.2 PBS_PBS, CD45.1 LPB_PBS, and CD45.2 LPS_PBS) and animals pre-exposed to LPS followed by HDM challenge regardless of macrophage ontogeny (CD45.1 or CD45.2 LPS_HDM) as there was a minimal cellular contribution to the unique IM2 sub-cluster (dark blue cells) by these samples (**Figure 5C**). Additionally, top gene analysis identified that the IM2 cluster was defined by expression of the *Chi3l3* (chitinase-like 3/Ym1), which has been previously associated with Th2, eosinophilic, and allergic airway responses (**Figure 5D**) (42–45). The Chi3l3 gene expression level was also most prominent in the CD45.2 PBS_HDM within the IM2 subcluster (*Chi3l3* ^hi^; dark blue cells). These findings suggest that *Chi3l3* ^hi^ CD45.2 IM2 cells uniquely and dynamically increased following HDM exposure and could potentially regulate allergic airway responses. Therefore, as opposed to AMØs, replacing embryonic-derived interstitial macrophages with bone marrow-derived ones is associated with dampening of HDM-induced allergic inflammation.

### Allergic response-associated pathways are enriched in embryonic-derived resident interstitial macrophages

**Figure 6:**
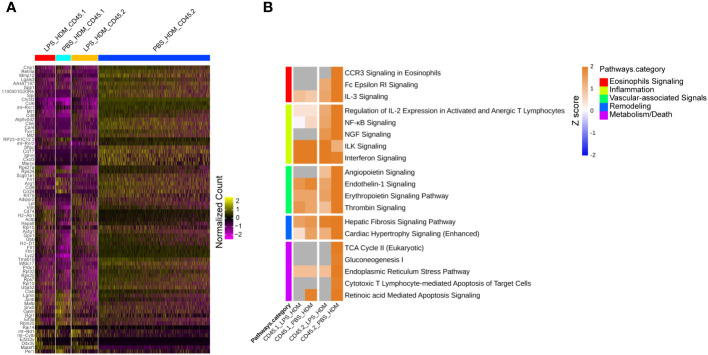
Tissue-resident interstitial macrophages from the IM2 cluster are enriched with allergic genes following HDM exposure and abrogated by LPS pre-HDM exposure. **(A)** Top 72 differentially regulated genes from the IM2 cluster from the following exposure conditions and macrophage origin: LPS_HDM in CD45.1 cells (LPS_HDM_CD45.1, red bar), PBS_HDM in CD45.1 cells (PBS_HDM_CD45.1, light blue bar), LPS_HDM in CD45.2 cells (LPS_HDM_CD45.2, orange bar) and PBS_HDM in CD45.2 cells (PBS_HDM_CD45.2, blue bar) expressed as a heatmap. **(B)** Ingenuity pathway analysis (Z score >2 or <2) of IM2 from CD45.1 (recruited) and CD45.2 cells (tissue-resident) based on exposure condition reveals that the IM2 cluster in CD45.2 (tissue-resident) cells is enriched for eosinophilic signaling (red bar), inflammation (yellow bar), vascular-associated signaling (green bar), remodeling (blue bar) and metabolism/cell death (purple bar). In the LPS_HDM exposed CD45.2 cluster, these pathways are reduced compared to the PBS_HDM group.

To better define the genetic pathways defining resident macrophages in the IM2 cluster, we performed an analysis based on ontogeny and exposure. A heatmap of top differentially expressed genes based on either the PBS_HDM or the LPS_HDM exposures and lineage (CD45.1 vs. CD45.2) of the IM2 cluster was generated (**Figure 6A**). This dataset revealed clear separation in the gene expression pattern in the CD45.2 PBS_HDM cells from other conditions (CD45.1 PBS_HDM, and CD45.1 *vs.* CD45.2 LPS_HDM). To clarify the pathways involved in this response, we performed an IPA pathway analysis to assess the potential functions of CD45.2 PBS_HDM IM2 cells. We focused on genes and pathways related to macrophage functions that could regulate allergic asthma, including processes related to eosinophil signaling, inflammation, vascular-associated signals, remodeling, and metabolism/death. Consistent with high *Chi3l3* expression, the CD45.2 PBS_HDM cells in IM2 were enriched for many macrophage-mediated inflammatory and remodeling processes relevant to allergic airway responses. These included those that regulate eosinophilic responses (e.g., CCR3 signaling in eosinophils, Fc Epsilon RI signaling, and IL-3) (**Figure 6B**). These pathways were downregulated in the LPS_HDM cell groups. In addition, we observed that CD45.1 bone marrow-derived macrophages (CD45.1_PBS_HDM) exhibited reduced allergic pathway activation compared to CD45.2 embryonic-derived macrophages (CD45.2_PBS_HDM), suggesting that replacement of CD45.2 embryonic-derived macrophages by CD45.1 bone marrow-derived macrophages reduces macrophage-derived allergic responses. While to a lesser degree than the difference observed across different ontogeny (CD45.2 *vs.* CD45.1), we also observed a reduction of allergic pathway signaling in CD45.2 embryonic-derived tissue-resident macrophages following LPS exposure (CD45.2_LPS_HDM) when compared to PBS exposed CD45.2 tissue-resident macrophages (CD45.2_PBS_HDM) suggesting a decrease of tissue-resident macrophage allergic potential in addition to replacement by CD45.1 cells (**Figure 6B**). These findings further indicate that there may be effects related to replacement by CD45.1 cells and the impact of prior exposure on the allergic potential of tissue-resident macrophages. We also observe that LPS pre-exposure leads to the downregulation of small numbers of inflammatory pathways (i.e., CCR5, IL-8, and thrombopoietin) in a lineage non-specific fashion (**Supplementary Figure S7**, Teal). As expected, there were upregulated pathways in response to HDM, which were shared across ontogeny or pre-exposure conditions (**Supplementary Figure S7**, Yellow). When taken together, these findings support that the ontogeny of interstitial macrophages is important for subsequent allergic responses to an allergen.

### IM2 macrophages co-express CD206 and CTSK and are located in the parenchyma of the terminal bronchioles of the PBS-HDM-treated animals

**Figure 7:**
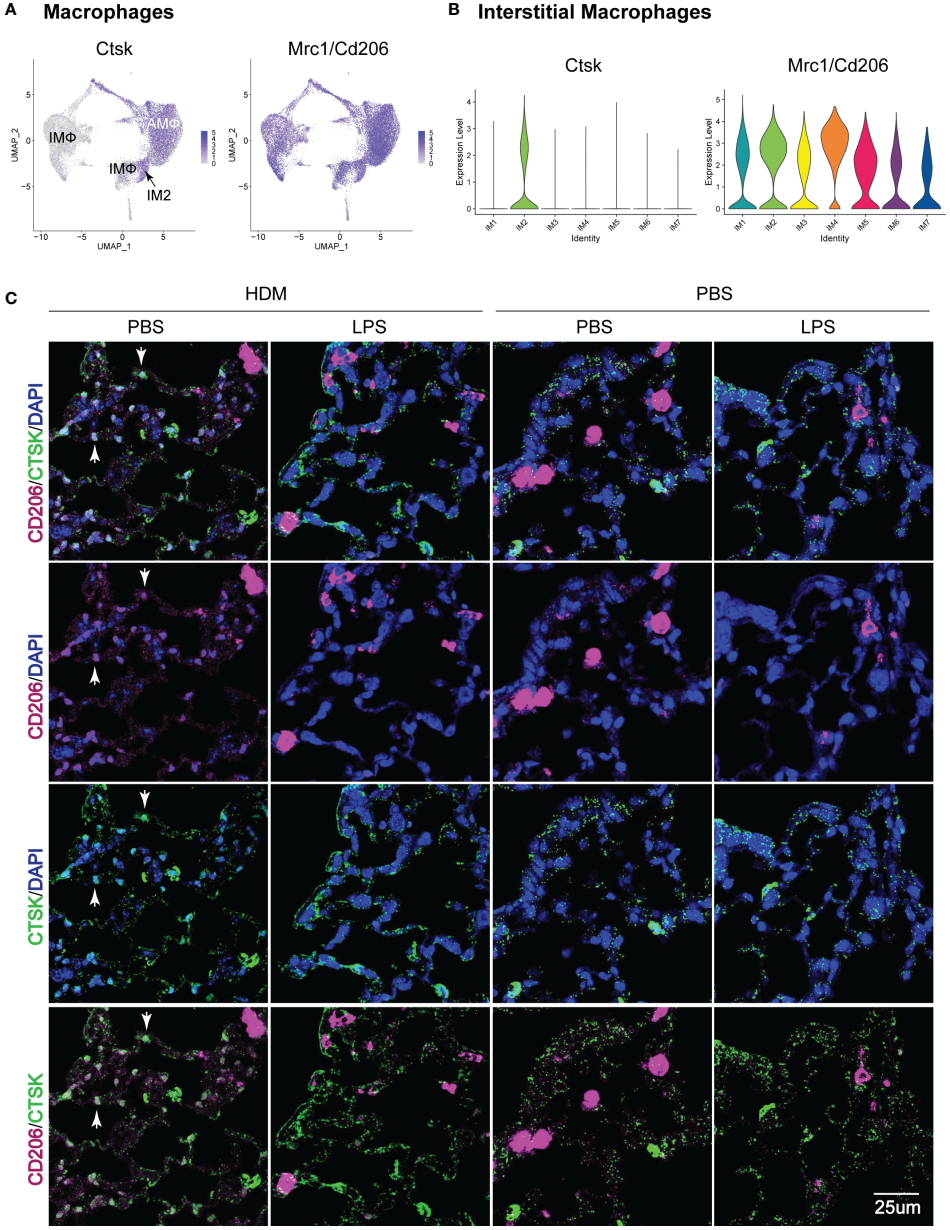
Immunofluorescence staining of IM2 cluster markers defines a subset of interstitial macrophages that localizes to the lung parenchyma adjacent to terminal bronchioles in PBS_HDM exposed mice. **(A)** Evaluation of gene expression patterns across macrophage clusters for pan-macrophage markers revealed broad expression of CD206 (mrc1). Cathepsin K (Ctsk) was widely expressed in alveolar macrophages, but in interstitial macrophages, Ctsk was identified as a marker primarily restricted to the CD45.2 IM2 cluster in the PBS_HDM exposed mice. **(B)** This was confirmed with violin plots of the IM clusters identifying broad Mrc1 expression but Ctsk expression unique to the IM2 cluster. **(C)** Immunofluorescence staining on lung tissue sections from PBS_PBS, PBS_HDM, LPS_PBS, and LPS_HDM exposed was performed for CD206 (pink, pan-macrophage marker), DAPI (blue, to identify nuclei) and Ctsk (green, CD45.2 IM2 cluster). In all exposure groups, CD206 staining was noted in airspace and parenchyma macrophages. In the PBS_HDM mice, CD206^+^ Ctsk^+^ and DAPI^+^ (white stained cells identified by arrowheads) cells were located in the lung parenchyma adjacent to terminal airways consistent with resident IM2 cluster cells. Co-localization of CD206 and Ctsk in macrophages was not appreciated in the PBS_PBS, LPS_PBS, or LPS_HDM groups supporting that these macrophages were unique to the HDM-exposed groups.

Given the identification of a unique IMØ cluster, which upregulated genes and pathways associated with HDM pulmonary allergic responses, we then attempted to define their location in lung tissue. By assessing the top gene expression of individual clusters based on exposure and ontogeny (**Supplementary Figure S8**), we identified *Mrc1*/*Cd206* (a macrophage marker) and *Ctsk* as uniquely co-expressed genes in the IM2 cluster (**Figures 7A, B**). Immunofluorescence analysis of lung tissue sections revealed a unique population of CD206^+^CTSK^+^ cells located in the parenchyma of the terminal bronchioles in the lungs of PBS_HDM treated mice (**Figure 7C**; **Supplementary Figure S9**). Consistent with our observations in ScRNAseq where, in PBS_HDM treated mice, a unique population of CD45.2 cells was evident and enriched for gene and pathways associated with allergic responses, the CD206^+^CTSK^+^ macrophages were only observed in alveolar regions adjacent to terminal bronchioles of PBS_HDM treated animals. Furthermore, CD206^+^CTSK^+^ double-positive cells were not observed in other conditions (**Figure 7C**, PBS_PBS, LPS_PBS, or LPS_HDM). This observation supports tissue locational evidence of a unique IMØ population following HDM exposure, central to regulating allergic airway responses.

## Discussion

The present study sought to address if prior environmental exposures lead to a persistent alteration of lung macrophage ontogeny, which regulates subsequent immune responses in allergic asthma. To address this question, we first demonstrated that the ontogeny of alveolar and interstitial macrophages is distinct, where AMØs appear to arise principally from fetal liver monocytes. In contrast, the majority of IMØs arise from yolk sac progenitors. We then demonstrated that LPS exposure induced the persistent replacement of embryonic-derived alveolar and interstitial macrophages with bone marrow-derived macrophages, supporting a change in the ontological landscape upon re-establishing homeostasis. Finally, we showed that LPS pre-exposed animals were protected from HDM-induced allergic airway disease. To clearly define processes and pathways underlying the protection from experimental allergic asthma and associated with alteration of macrophage ontogenetic landscape, we performed single-cell RNA-sequencing from flow-sorted cells based on ontogeny. We identified a specific IMØ subset arising from embryonic-derived IMØs, located adjacent to terminal bronchioles, and only present in HDM treated without the LPS pre-exposure. This IMØ subset had increased gene expression related to generating allergic airway responses. Overall, this data supports that the ontogeny of macrophages, particularly IMØs, is important for allergic airway disease and suggests that approaches to alter macrophage ontogeny may be relevant as a potential prophylactic therapy for the development of asthma.

Our focus on ontogeny was due to the observation that lung macrophages exhibit different ontogenies (*i.e.*, embryonic-derived or bone marrow-derived). Historically, it was assumed that lung macrophages were either derived from bone marrow origin or largely replaced over the lifespan by bone marrow precursors, thereby limiting the consideration of tissue-resident ontogeny as an effect modifier in immune responses. Recent studies have challenged this notion using detailed lineage tracing and parabiosis system demonstrating, under homeostatic conditions, AMØs are embryonic-derived and exhibit self-renewal. Furthermore, these AMØs arise from fetal liver monocyte precursors (46, 47). IMØ ontogeny has also been defined, though with less overall clarity. Early studies suggest IMØs are yolk sac-derived cells arising around E8.5 or earlier (27, 28). Using a *runx1* reporter to define extra-embryonic yolk sac-derived cells, Tan and colleagues (31) described two populations of macrophages in the interstitium during the early post-natal period: primitive yolk sac-derived and bone marrow-derived. They observed that bone marrow-derived intermediates were gradually recruited to the interstitial space and increased in number over the early post-natal period. In these studies, it was unclear if the bone marrow-derived macrophages eventually overtake yolk sac-derived macrophages as animals age. Subsequently, in adult murine lungs, Chakarov et al. used single-cell RNA-seq and S100A4-EYFP lineage labeling to identify two monocyte-derived IMØ populations (defined by differential expression of Lyve1 and MHCII) located either around nerve endings or the vasculature (5). Though they tracked bone marrow-derived IMØ using an S100A4-EYFP reporter, they did not examine the presence of macrophages of embryonic origins. Alternatively, Gibbings et al. identified three distinct IMØ populations by flow cytometry (based on differential expression of Lyve1, MHCII, and CCR2) and demonstrated that these IMØ had differing gene expression (26). This observation was confirmed by Dick et al., using single-cell transcriptomic analyses followed by lineage tracing, describing three populations of tissue-resident pulmonary IMØs (38). In contrast to Chakarov et al., they defined a Lyve1+ population arising from both yolk sac and fetal liver monocytes, an MHCII+ population from mixed origins, and a CCR2+ population continuously replenished from circulating monocytes. Our findings in the present study are largely consistent with those of Dick et al., though we observed that IMØ were mainly yolk-sac derived, without clear derivation from fetal liver monocytes. This discrepancy may have to do with the timing of our lineage induction at E7.5 and E13.5 to distinguish yolk-sac vs. fetal monocyte origins, as opposed to E14.5 and E19.5 in other reports where distinct embryonic origins cannot be distinguished using Cx3cr1^cre/ER^-based lineage tracing system. In addition, we demonstrated that the lineage labeled macrophages were maintained through adulthood, supporting that, under uninjured conditions, these embryonic-derived IMØs are maintained in the adult. Therefore, our study added to the prior literature by defining distinct ontogenies of lung macrophages preserved into adulthood in the unchallenged state.

Despite defining macrophage ontogeny at a steady state in the uninjured mouse, less is understood about how ontogenetic landscape changes with prior lung injury and how these alterations impact subsequent immune responses. Previous research has focused on differential effects of ontogeny following acute lung injury, particularly differences between tissue-resident and monocyte-derived (i.e., bone marrow-derived) AMØs (3). In models of acute lung injury with bleomycin, asbestosis, influenza, and LPS (8–10, 48–51), bone marrow-derived AMØ are recruited to the lung to direct inflammatory and/or fibrotic responses. In cases of severe lung injury, these bone marrow-derived AMØs can persist after the resolution of lung injury, becoming chimeric with tissue-resident AMØs. Over time and without additional injury, these bone marrow-derived AMØ assume a genetic profile similar to embryonic-derived AMØs (8, 10, 49). This process likely reflects an “empty” niche, generated after severe lung injury, where bone marrow-derived AMØ can take up residence. Consistent with this, repopulation with yolk-sac, fetal monocyte, or bone-marrow progenitors into Csf2rb-/- mice, which have an empty developmental “niche” for alveolar macrophages, leads to AMØs with nearly identical gene expression patterns independent of the progenitor source (40).

Though the effects of ontogeny in acute lung injury have been defined, less is known about how altered ontogeny from prior injury events that have returned to homeostasis impacts subsequent response. This is particularly relevant as prior exposures or events during lung development or postnatally can change immune cell composition in tissues and thereby have the potential to alter their response to subsequent immune stimuli. We addressed this knowledge gap by performing LPS exposure to generate lung inflammation and direct turnover of embryonic-derived AMØs and IMØs. We confirmed the return to homeostasis by demonstrating minimal differences in the immune profile through histologic analyses, flow cytometry, and cytokine profiles between the PBS_PBS and the LPS_PBS immune cells, and physiologic response to methacholine challenge (**Figure 2**; **Supplementary Figures S3**, **4**). This experimental design was utilized to determine the impact of embryonic-derived lung macrophage replacement without the complicated effects of LPS-induced acute inflammation. When we exposed mice to HDM under conditions where embryonic-derived macrophages had been replaced with bone marrow-derived AMØ and IMØs, we observed protection from HDM-induced allergic inflammation, mucus hyperplasia, and airway hyperresponsiveness (**Figures 2C–F**). This data supports that prior exposures can alter subsequent lung responses and highlights a need for understanding how these prior exposures impact the macrophage compositional profile and function when the lung returns to homeostasis.

Our observations are consistent with recent literature demonstrating that prior murine lung viral infection with murine herpesvirus 4 or murine-adapted influenza A attenuates allergic airway inflammation or subsequent pneumovirus infection (52–54). This supports a common theme: prior innate immune activation can attenuate subsequent allergic airway responses. Similar to our observation of AMØ turnover following LPS (**Figure 2B**), Machiels et al. identified replacement of AMØs following murine herpesvirus 4 infections (52). Furthermore, they reported that replacing AMØs drove reduced allergic responses. Though our studies also observed replacement of AMØs following LPS exposure, our single-cell RNA sequencing studies did not reveal a dominant AMØ cluster in the PBS_HDM exposure groups with upregulated allergic asthma-associated genes (**Figure 4**; **Supplementary Figures S5**, **6**). We also did not observe strong effects of ontogeny on AMØ transcriptomic profile following HDM exposure (CD45.1 vs. CD45.2). Despite a lack of an HDM effect, prior exposure to LPS significantly alters AMØ transcriptomic profile, suggesting the potential for altered subsequent AMØ responses to immune stimuli (*e.g.*, trained memory effects), which could be evaluated in future studies.

Despite an observed impact on AMØs, Machiels et al. did not explore the impact on IMØs, whose origin is also altered by respiratory viral infections (55) and can impact allergic airway responses (6, 56, 57). Consistent with these IMØ-focused studies, our single-cell RNA sequencing studies identified a population of embryonic-derived IMØs (IM2 cluster, **Figure 6**) that upregulated pathways and processes associated with allergic asthma responses. In addition, using the gene expression data from this cluster, we defined the tissue location of these cells within the lung interstitial spaces adjacent to terminal bronchioles (**Figure 7B**; **Supplementary Figure 9**). This data suggests that embryonic-derived IMØs promote allergic airway responses, and replacing these embryonic-derived IMØs with bone marrow-derived IMØs is associated with reduced allergic responses. In addition to the observed replacement of embryonic-derived IMØs with bone marrow-derived IMØs, we also observed an impact of the prior LPS-induced inflammation on the programming of embryonic-derived IMØs in response to HDM challenge, consistent with the concept of trained immunity (**Supplementary Figure S7**) (58). This conclusion was evidenced in the IM2 cluster from the LPS_HDM_CD45.2 exposure conditions, where we observed reduced allergic programming relative to the IM2 cluster from the PBS_HDM_CD45.2 exposure conditions. Overall, our data suggests complex dynamics interplay that drives allergic airway disease, involving interstitial macrophage ontogeny and trained immunity by prior lung injury.

To our knowledge, this study is the first description of how prior exposures shape the subsequent ontogenetic landscape of pulmonary alveolar and interstitial macrophages and their functional profile. The observation has important implications for understanding human lung immunity. Humans do not exist in a sterile environment but experience environmental challenges over their lifespan that can impact allergic responses. Consistent with this observation, prior environmental LPS exposure, linking to the hygiene hypothesis, has been associated with reduced allergic sensitization and asthma (59–61). Stein and colleagues identified differing asthma prevalence in two Amish cohorts based on environmental conditions (59). They characterized dust from the homes of the two Amish cohorts, identifying that they differed based on their LPS content. When administered with ovalbumin to rodents, the low asthma prevalence/high LPS content dust failed to elicit allergic inflammation and airway hyperresponsiveness. This suggests a protective effect of LPS exposure in allergic airway responses. The specific mechanisms driving these effects were not defined in this study, but the authors did identify that it required innate immune signaling via MyD88. Interestingly, MyD88 is a critical signaling node of lung macrophages, highlighting the potential of macrophages as a driver of reductions in allergy associated with the hygiene hypothesis. Our data support that IMØs may exert a central role in these responses. Therefore, understanding the dynamics of prior exposure to macrophage functions in homeostasis and response to subsequent challenges is critical. Though not explicitly addressed in this initial study, future studies will also be required to assess the differential impact of replacement versus memory of prior exposures on allergic airway responses and if these regulate the responses attributed to the hygiene hypothesis.

Some limitations should be considered in the present study. Though our study focused on macrophages, we acknowledge that T cells are an essential effect modifier of allergic airway responses (62), they exhibit crosstalk with macrophages in this context (63), and their function can be altered by LPS exposure. Similarly, we acknowledge that epithelial cells are important for allergic responses and can be modified by LPS exposure (64, 65). Though we did not observe gross structural differences in epithelial composition and morphology following LPS (**Supplementary Figure S2**), our studies did not explicitly examine epithelial and other lung structural cells. T cell and epithelial-specific effects on allergic responses will need to be considered in future studies and analyses. We also acknowledge that the present study does not define a causal role for the IM2 cluster. We demonstrated that the IM2 cluster upregulates genes related to allergic airway responses and is located within the tissue adjacent to terminal airway structures. However, as the methods for the targeted examination of this specific cluster are not presently available, we could not demonstrate clear causality for this IMØ subset in promoting allergic responses. Despite efforts to ablate IMØ by chemical or genetic methods, we were not able to target IMØ with specificity or without significant associated inflammation (data not shown). Future efforts will need to identify targeted strategies that impact IMØ subsets for causal studies.

In summary, we defined distinct ontogenies of AMØ and IMØ. Their embryonic ontogeny is maintained in adult mice but can be altered by prior LPS exposure. Furthermore, LPS exposure attenuates subsequent HDM-mediated allergic airway responses. This effect is associated with altering IMØ ontogenetic composition and genetic programming. Overall, this study suggests that lung macrophage ontogeny is important for allergic airway responses and highlights a potential role for IMØ ontogeny in these responses.

## Data availability statement

The datasets presented in this study can be found in online repositories. The names of the repository/repositories and accession number(s) can be found below: https://dataview.ncbi.nlm.nih.gov/object/PRJNA1049857?reviewer=b0qaf0lln37bmj86789uc01oj9, PRJNA1049857.

## Ethics statement

The animal study was approved by School of Medicine, Duke University. The study was conducted in accordance with the local legislation and institutional requirements.

## Author contributions

RT: Writing – review & editing, Writing – original draft, Visualization, Validation, Software, Resources, Methodology, Investigation, Funding acquisition, Formal analysis. AB: Writing – review & editing, Data curation. YM: Writing – review & editing, Methodology, Data curation. YK: Writing – review & editing, Methodology, Data curation. AV: Writing – review & editing, Methodology, Formal analysis, Data curation. VC: Writing – review & editing, Methodology, Formal analysis, Data curation. JC: Writing – review & editing, Methodology, Formal analysis, Data curation. RC: Writing – review & editing, Methodology, Formal analysis, Data curation. HF: Writing – review & editing, Writing – original draft, Visualization, Software, Methodology, Formal analysis. PT: Writing – review & editing, Methodology, Data curation. JI: Writing – review & editing, Methodology, Formal analysis, Data curation. MG: Writing – review & editing, Supervision, Resources, Methodology. LQ: Writing – review & editing, Resources, Methodology, Data curation. YY: Writing – review & editing, Writing – original draft, Visualization, Validation, Supervision, Software, Resources, Project administration, Methodology, Investigation, Funding acquisition, Formal analysis, Data curation, Conceptualization.

## References

[1] Hou F Xiao K Tang L Xie L. Diversity of macrophages in lung homeostasis and diseases. Front Immunol. (2021) 12:753940. doi: 10.3389/fimmu.2021.753940 34630433 PMC8500393

[2] Ogger PP Byrne AJ. Macrophage metabolic reprogramming during chronic lung disease. Mucosal Immunol. (2021) 14:282–95. doi: 10.1038/s41385-020-00356-5 PMC7658438 33184475

[3] Joshi N Walter JM Misharin AV. Alveolar macrophages. Cell Immunol. (2018) 330:86–90. doi: 10.1016/j.cellimm.2018.01.005 29370889

[4] Evren E Ringqvist E Willinger T. Origin and ontogeny of lung macrophages: from mice to humans. Immunology. (2020) 160:126–38. doi: 10.1111/imm.13154 PMC7218405 31715003

[5] Chakarov S Lim HY Tan L Lim SY See P Lum J. Two distinct interstitial macrophage populations coexist across tissues in specific subtissular niches. Science. (2019) 363. doi: 10.1126/science.aau0964 30872492

[6] Sabatel C Radermecker C Fievez L Paulissen G Chakarov S Fernandes C. Exposure to bacterial CpG DNA protects from airway allergic inflammation by expanding regulatory lung interstitial macrophages. Immunity. (2017) 46:457–73. doi: 10.1016/j.immuni.2017.02.016 28329706

[7] Jenkins SJ Allen JE. The expanding world of tissue-resident macrophages. Eur J Immunol. (2021) 51:1882–96. doi: 10.1002/eji.202048881 34107057

[8] Misharin AV Morales-Nebreda L Reyfman PA Cuda CM Walter JM McQuattie-Pimentel AC. Monocyte-derived alveolar macrophages drive lung fibrosis and persist in the lung over the life span. J Exp Med. (2017) 214:2387–404. doi: 10.1084/jem.20162152 PMC5551573 28694385

[9] Mould KJ Barthel L Mohning MP Thomas SM McCubbrey AL Danhorn T. Cell Origin Dictates Programming of Resident versus Recruited Macrophages during Acute Lung Injury. Am J Respir Cell Mol Biol. (2017) 57:294–306. doi: 10.1165/rcmb.2017-0061OC 28421818 PMC5625228

[10] McQuattie-Pimentel AC Ren Z Joshi N Watanabe S Stoeger T Chi M. The lung microenvironment shapes a dysfunctional response of alveolar macrophages in aging. J Clin Invest. (2021) 131. doi: 10.1172/JCI140299 PMC7919859 33586677

[11] Yu YA Malakhau Y Yu CA Phelan SJ Cumming RI Kan MJ. Nonclassical monocytes sense hypoxia, regulate pulmonary vascular remodeling, and promote pulmonary hypertension. J Immunol. (2020) 204(6):1474–85. doi: 10.4049/jimmunol.1900239 PMC7065976 31996456

[12] Cyphert-Daly JM Yang Z Ingram JL Tighe RM Que LG. Physiologic response to chronic house dust mite exposure in mice is dependent on lot characteristics. J Allergy Clin Immunol. (2019) 144:1428–32.e1428. doi: 10.1016/j.jaci.2019.07.019 31369802 PMC6842440

[13] Hantos Z Adamicza A Govaerts E Daroczy B. Mechanical impedances of lungs and chest wall in the cat. J Appl Physiol. (1992) 73:427–33. doi: 10.1152/jappl.1992.73.2.427 1399961

[14] Irvin CG Bates JH. Measuring the lung function in the mouse: the challenge of size. Respir Res. (2003) 4:4. doi: 10.1186/rr199 12783622 PMC184039

[15] Yu YR O'Koren EG Hotten DF Kan MJ Kopin D Nelson ER. A protocol for the comprehensive flow cytometric analysis of immune cells in normal and inflamed murine non-lymphoid tissues. PLoS One. (2016) 11:e0150606. doi: 10.1371/journal.pone.0150606 26938654 PMC4777539

[16] Birukova A Cyphert-Daly J Cumming RI Yu YR Gowdy KM Que LG. Sex modifies acute ozone-mediated airway physiologic responses. Toxicological Sci. (2019) 169:499–510. doi: 10.1093/toxsci/kfz056 PMC6542336 30825310

[17] Tighe RM Redente EF Yu YR Herold S Sperling AI Curtis JL. Improving the quality and reproducibility of flow cytometry in the lung. An official American thoracic society workshop report. Am J Respir Cell Mol Biol. (2019) 61:150–61. doi: 10.1165/rcmb.2019-0191ST PMC6670040 31368812

[18] Lin R Degan S Theriot BS Fischer BM Strachan RT Liang J. Chronic treatment in *vivo* with beta-adrenoceptor agonists induces dysfunction of airway beta(2) -adrenoceptors and exacerbates lung inflammation in mice. Br J Pharmacol. (2012) 165:2365–77. doi: 10.1111/j.1476-5381.2011.01725.x PMC3413869 22013997

[19] Hollingsworth JW Theriot BS Li Z Lawson BL Sunday M Schwartz DA. Both hematopoietic-derived and non-hematopoietic-derived {beta}-arrestin-2 regulates murine allergic airway disease. Am J Respir Cell Mol Biol. (2010) 43:269–75. doi: 10.1165/rcmb.2009-0198OC PMC2933545 19805483

[20] Kersey PJ Staines DM Lawson D Kulesha E Derwent P Humphrey JC. Ensembl Genomes: an integrative resource for genome-scale data from non-vertebrate species. Nucleic Acids Res. (2012) 40:D91–97. doi: 10.1093/nar/gkr895 PMC3245118 22067447

[21] Macosko EZ Basu A Satija R Nemesh J Shekhar K Goldman M. Highly parallel genome-wide expression profiling of individual cells using nanoliter droplets. Cell. (2015) 161:1202–14. doi: 10.1016/j.cell.2015.05.002 PMC4481139 26000488

[22] McCarthy DJ Campbell KR Lun AT Wills QF. Scater: pre-processing, quality control, normalization and visualization of single-cell RNA-seq data in R. Bioinformatics. (2017) 33:1179–86. doi: 10.1093/bioinformatics/btw777 PMC5408845 28088763

[23] Stuart T Butler A Hoffman P Hafemeister C Papalexi E Mauck WM 3rd. Comprehensive integration of single-cell data. Cell. (2019) 177:1888–902.e1821. doi: 10.1016/j.cell.2019.05.031 31178118 PMC6687398

[24] Aran D Looney AP Liu L Wu E Fong V Hsu A. Reference-based analysis of lung single-cell sequencing reveals a transitional profibrotic macrophage. Nat Immunol. (2019) 20:163–72. doi: 10.1038/s41590-018-0276-y PMC6340744 30643263

[25] Heng TS Painter MW Immunological Genome Project C. The Immunological Genome Project: networks of gene expression in immune cells. Nat Immunol. (2008) 9:1091–4. doi: 10.1038/ni1008-1091 18800157

[26] Gibbings SL Thomas SM Atif SM McCubbrey AL Desch AN Danhorn T. Three unique interstitial macrophages in the murine lung at steady state. Am J Respir Cell Mol Biol. (2017) 57:66–76. doi: 10.1165/rcmb.2016-0361OC 28257233 PMC5516280

[27] Gomez Perdiguero E Klapproth K Schulz C Busch K Azzoni E Crozet L. Tissue-resident macrophages originate from yolk-sac-derived erythro-myeloid progenitors. Nature. (2015) 518:547–51. doi: 10.1038/nature13989 PMC5997177 25470051

[28] Stremmel C Schuchert R Wagner F Thaler R Weinberger T Pick R. Yolk sac macrophage progenitors traffic to the embryo during defined stages of development. Nat Commun. (2018) 9:75. doi: 10.1038/s41467-018-06065-9 29311541 PMC5758709

[29] Hoeffel G Chen J Lavin Y Low D Almeida FF See P. C-Myb(+) erythro-myeloid progenitor-derived fetal monocytes give rise to adult tissue-resident macrophages. Immunity. (2015) 42:665–78. doi: 10.1016/j.immuni.2015.03.011 PMC4545768 25902481

[30] Hashimoto D Chow A Noizat C Teo P Beasley MB Leboeuf M. Tissue-resident macrophages self-maintain locally throughout adult life with minimal contribution from circulating monocytes. Immunity. (2013) 38:792–804. doi: 10.1016/j.immuni.2013.04.004 23601688 PMC3853406

[31] Tan SY Krasnow MA. Developmental origin of lung macrophage diversity. Development. (2016) 143:1318–27. doi: 10.1242/dev.129122 PMC4852511 26952982

[32] Scott CL Zheng F De Baetselier P Martens L Saeys Y De Prijck S. Bone marrow-derived monocytes give rise to self-renewing and fully differentiated Kupffer cells. Nat Commun. (2016) 7:10321. doi: 10.1038/ncomms10321 26813785 PMC4737801

[33] Louwe PA Badiola Gomez L Webster H Perona-Wright G Bain CC Forbes SJ. Recruited macrophages that colonize the post-inflammatory peritoneal niche convert into functionally divergent resident cells. Nat Commun. (2021) 12:1770. doi: 10.1038/s41467-021-21778-0 33741914 PMC7979918

[34] Yona S Kim KW Wolf Y Mildner A Varol D Breker M. Fate mapping reveals origins and dynamics of monocytes and tissue macrophages under homeostasis. Immunity. (2013) 38:79–91. doi: 10.1016/j.immuni.2012.12.001 23273845 PMC3908543

[35] Guilliams M Scott CL. Does niche competition determine the origin of tissue-resident macrophages? Nat Rev Immunol. (2017) 17:451–60. doi: 10.1038/nri.2017.42 28461703

[36] Ginhoux F Guilliams M. Tissue-resident macrophage ontogeny and homeostasis. Immunity. (2016) 44:439–49. doi: 10.1016/j.immuni.2016.02.024 26982352

[37] Ginhoux F Greter M Leboeuf M Nandi S See P Gokhan S. Fate mapping analysis reveals that adult microglia derive from primitive macrophages. Science. (2010) 330:841–5. doi: 10.1126/science.1194637 PMC3719181 20966214

[38] Dick SA Wong A Hamidzada H Nejat S Nechanitzky R Vohra S. Three tissue resident macrophage subsets coexist across organs with conserved origins and life cycles. Sci Immunol. (2022) 7:eabf7777. doi: 10.1126/sciimmunol.abf7777 34995099

[39] Gibbings SL Goyal R Desch AN Leach SM Prabagar M Atif SM. Transcriptome analysis highlights the conserved difference between embryonic and postnatal-derived alveolar macrophages. Blood. (2015) 126:1357–66. doi: 10.1182/blood-2015-01-624809 PMC4566811 26232173

[40] van de Laar L Saelens W De Prijck S Martens L Scott CL Van Isterdael G. Yolk sac macrophages, fetal liver, and adult monocytes can colonize an empty niche and develop into functional tissue-resident macrophages. Immunity. (2016) 44:755–68. doi: 10.1016/j.immuni.2016.02.017 26992565

[41] Beattie L Sawtell A Mann J Frame TCM Teal B de Labastida Rivera F. Bone marrow-derived and resident liver macrophages display unique transcriptomic signatures but similar biological functions. J Hepatol. (2016) 65:758–68. doi: 10.1016/j.jhep.2016.05.037 PMC5028381 27262757

[42] Wan S Sun X Wu F Yu Z Wang L Lin D. Chi3l3: a potential key orchestrator of eosinophil recruitment in meningitis induced by Angiostrongylus cantonensis. J Neuroinflamm. (2018) 15:31. doi: 10.1186/s12974-018-1071-2 PMC5796390 29391024

[43] Zhao T Su Z Li Y Zhang X You Q. Chitinase-3 like-protein-1 function and its role in diseases. Signal Transduct Target Ther. (2020) 5:201. doi: 10.1038/s41392-020-00303-7 32929074 PMC7490424

[44] Abdelaziz MH Abdelwahab SF Wan J Cai W Huixuan W Jianjun C. Alternatively activated macrophages; a double-edged sword in allergic asthma. J Transl Med. (2020) 18:58. doi: 10.1186/s12967-020-02251-w 32024540 PMC7003359

[45] Draijer C Robbe P Boorsma CE Hylkema MN Melgert BN. Dual role of YM1+ M2 macrophages in allergic lung inflammation. Sci Rep. (2018) 8:5105. doi: 10.1038/s41598-018-23269-7 29572536 PMC5865212

[46] Guilliams M De Kleer I Henri S Post S Vanhoutte L De Prijck S. Alveolar macrophages develop from fetal monocytes that differentiate into long-lived cells in the first week of life via GM-CSF. J Exp Med. (2013) 210:1977–92. doi: 10.1084/jem.20131199 PMC3782041 24043763

[47] Sheng J Ruedl C Karjalainen K. Most tissue-resident macrophages except microglia are derived from fetal hematopoietic stem cells. Immunity. (2015) 43:382–93. doi: 10.1016/j.immuni.2015.07.016 26287683

[48] Joshi N Watanabe S Verma R Jablonski RP Chen CI Cheresh P. A spatially restricted fibrotic niche in pulmonary fibrosis is sustained by M-CSF/M-CSFR signalling in monocyte-derived alveolar macrophages. Eur Respir J. (2020) 55. doi: 10.1183/13993003.00646-2019 PMC6962769 31601718

[49] Aegerter H Kulikauskaite J Crotta S Patel H Kelly G Hessel EM. Influenza-induced monocyte-derived alveolar macrophages confer prolonged antibacterial protection. Nat Immunol. (2020) 21:145–57. doi: 10.1038/s41590-019-0568-x PMC6983324 31932810

[50] Janssen WJ Barthel L Muldrow A Oberley-Deegan RE Kearns MT Jakubzick C. Fas determines differential fates of resident and recruited macrophages during resolution of acute lung injury. Am J Respir Crit Care Med. (2011) 184:547–60. doi: 10.1164/rccm.201011-1891OC PMC3175550 21471090

[51] Lin KL Suzuki Y Nakano H Ramsburg E Gunn MD. CCR2+ monocyte-derived dendritic cells and exudate macrophages produce influenza-induced pulmonary immune pathology and mortality. J Immunol. (2008) 180:2562–72. doi: 10.4049/jimmunol.180.4.2562 18250467

[52] Machiels B Dourcy M Xiao X Javaux J Mesnil C Sabatel C. A gammaherpesvirus provides protection against allergic asthma by inducing the replacement of resident alveolar macrophages with regulatory monocytes. Nat Immunol. (2017) 18:1310–20. doi: 10.1038/ni.3857 29035391

[53] Wu Q Jorde I Kershaw O Jeron A Bruder D Schreiber J. Resolved influenza A virus infection has extended effects on lung homeostasis and attenuates allergic airway inflammation in a mouse model. Microorganisms. (2020) 8. doi: 10.3390/microorganisms8121878 PMC7761027 33260910

[54] Gilliaux G Desmecht D. Gammaherpesvirus alters alveolar macrophages according to the host genetic background and promotes beneficial inflammatory control over pneumovirus infection. Viruses. (2022) 14. doi: 10.3390/v14010098 PMC8777918 35062301

[55] Corry J Kettenburg G Upadhyay AA Wallace M Marti MM Wonderlich ER. Infiltration of inflammatory macrophages and neutrophils and widespread pyroptosis in lung drive influenza lethality in nonhuman primates. PloS Pathog. (2022) 18:e1010395. doi: 10.1371/journal.ppat.1010395 35271686 PMC8939778

[56] Bedoret D Wallemacq H Marichal T Desmet C Quesada Calvo F Henry E. Lung interstitial macrophages alter dendritic cell functions to prevent airway allergy in mice. J Clin Invest. (2009) 119:3723–38. doi: 10.1172/JCI39717 PMC2786798 19907079

[57] Fu Y Wang J Zhou B Pajulas A Gao H Ramdas B. An IL-9-pulmonary macrophage axis defines the allergic lung inflammatory environment. Sci Immunol. (2022) 7:eabi9768. doi: 10.1126/sciimmunol.abi9768 35179949 PMC8991419

[58] Netea MG Dominguez-Andres J Barreiro LB Chavakis T Divangahi M Fuchs E. Defining trained immunity and its role in health and disease. Nat Rev Immunol. (2020) 20:375–88. doi: 10.1038/s41577-020-0285-6 PMC7186935 32132681

[59] Stein MM Hrusch CL Gozdz J Igartua C Pivniouk V Murray SE. Innate immunity and asthma risk in Amish and Hutterite farm children. New Engl J Med. (2016) 375:411–21. doi: 10.1056/NEJMoa1508749 PMC5137793 27518660

[60] von Mutius E. The microbial environment and its influence on asthma prevention in early life. J Allergy Clin Immunol. (2016) 137:680–9. doi: 10.1016/j.jaci.2015.12.1301 26806048

[61] Braun-Fahrlander C Riedler J Herz U Eder W Waser M Grize L. Environmental exposure to endotoxin and its relation to asthma in school-age children. New Engl J Med. (2002) 347:869–77. doi: 10.1056/NEJMoa020057 12239255

[62] Larche M Robinson DS Kay AB. The role of T lymphocytes in the pathogenesis of asthma. J Allergy Clin Immunol. (2003) 111:450–463; quiz 464. doi: 10.1067/mai.2003.169 12642820

[63] Zhu X Cui J Yi Qin J Tulake W Teng F. The role of T cells and macrophages in asthma pathogenesis: A new perspective on mutual crosstalk. Mediators Inflammation. (2020) 2020:7835284. doi: 10.1155/2020/7835284 PMC7453253 32922208

[64] Jakwerth CA Ordovas-Montanes J Blank S Schmidt-Weber CB Zissler UM. Role of respiratory epithelial cells in allergic diseases. Cells. (2022) 11. doi: 10.3390/cells11091387 PMC9105716 35563693

[65] Skerrett SJ Liggitt HD Hajjar AM Ernst RK Miller SI Wilson CB. Respiratory epithelial cells regulate lung inflammation in response to inhaled endotoxin. Am J Physiol Lung Cell Mol Physiol. (2004) 287:L143–152. doi: 10.1152/ajplung.00030.2004 15047567

