# Supplementary material for: Altered ontogeny and transcriptomic signatures of tissue-resident pulmonary interstitial macrophages ameliorate allergic airway hyperresponsiveness
Source: Front Immunol. 2024 Jun 25;15:1371764. doi: 10.3389/fimmu.2024.1371764 (PMC11231371; doi:10.3389/fimmu.2024.1371764)
Supplement: Supplementary file 1 [file DataSheet_1.pdf]

Online Data Supplement

Allergic Asthma Responses Are Dependent on Interstitial Macrophage Ontogeny

Robert M. Tighe, Anastasiya Birukova, Yuryi Malakhau, Aaron T. Vose, Vidya Chandramohan, Jaime M. Cyphert-Daly, R. Ian Cumming, Alexander V. Misharin, Yoshihiko Kobayashi, Purushothama R. Tata, Jennifer L. Ingram, Michael D. Gunn, Loretta G. Que, Yen-Rei A. Yu

Table S1 - Flow Cytometry Antibodies in base panel and additional markers to confirm cell type

Antibody	Clone	Dilutions	Fluorochrome	Company	Catalog #
CD11b	M1/70	1:50	APC-Cy7	BD Biosciences	557657
CD11c	HL3	1:100	BV785	BD Biosciences	563735
CD24	M1/69	1:800	BV711	BD Biosciences	563450
CD45	30-F11	1:500	BV605	Biolegend	103139
CD49b	DX5	1:100	PE	eBioscience	12-5971-83
CD64	X54-5/7.1	1:200	BV421	Biolegend	139309
IA/IE	M5/114.15.2	1:1500	BV650	BD Biosciences	563415
Ly6C	HK1.4	1:200	PerCP-Cy5.5	eBioscience	45-5932
Ly6G	1A8	1:200	AF700	BD Biosciences	561236
Zombie Yellow	NA	1:500	ex:405nm/em:572nm	Biolegend	423104
SiglecF	E50-2440	1:500	PE-CF594	BD Biosciences	562757
CD3	145-2C11	1:100	AF488	Biolegend	100321
CD31	MEC 13.3	1:400	PerCP-Cy5.5	Biolegend	562861
CD117	2B8	1:100	BV510	Biolegend	105839
B220	RA3-6B12	1:100	AF647	Biolegend	103226
F4/80	BM8	1:400	PE-Cy7	eBioscience	25-4801
FceR1	MAR-1	1:100	PE-eF610	eBioscience	61-5898-82

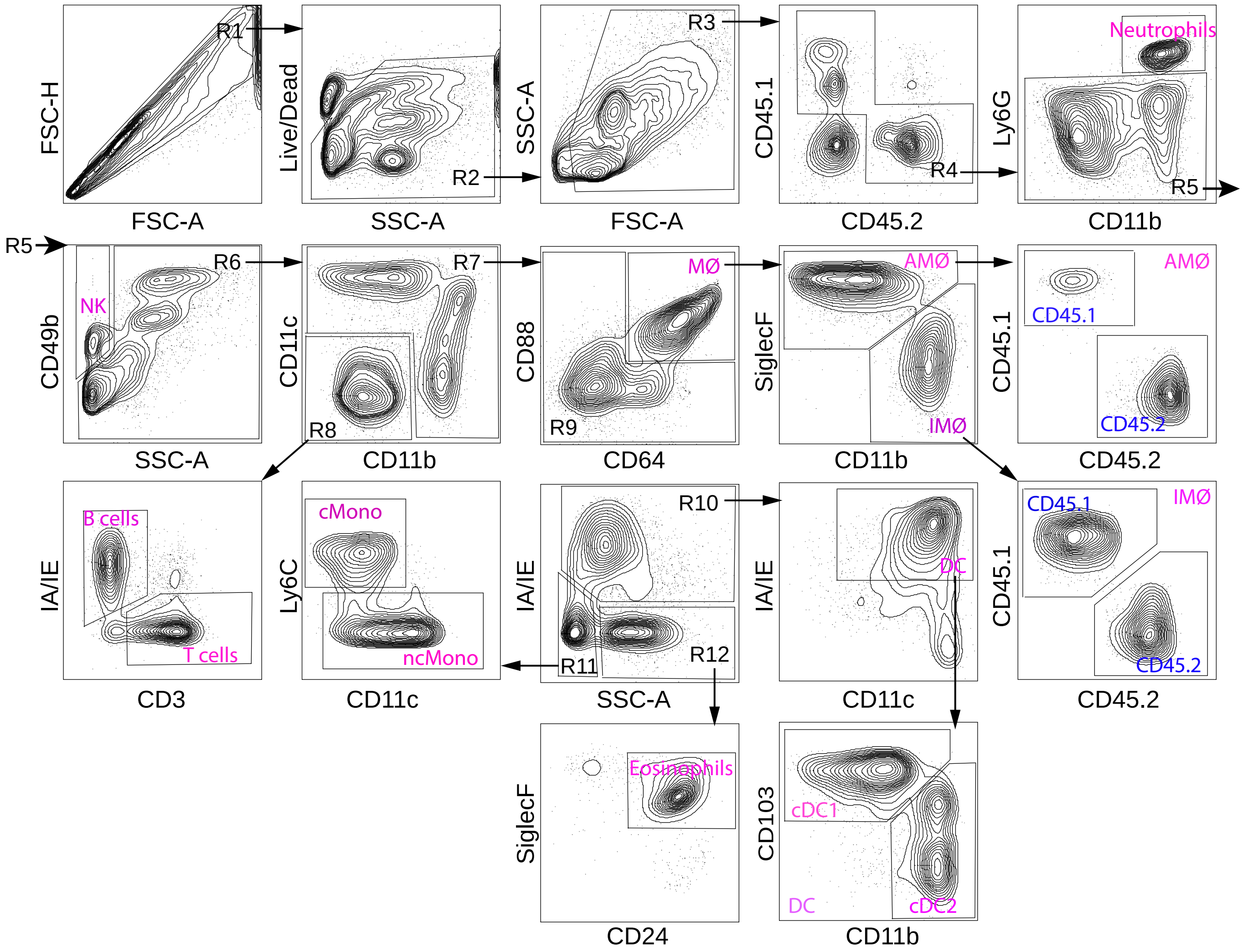

Supplemental Figure S1. *Flow cytometry gating strategy to identify immune cell populations.*

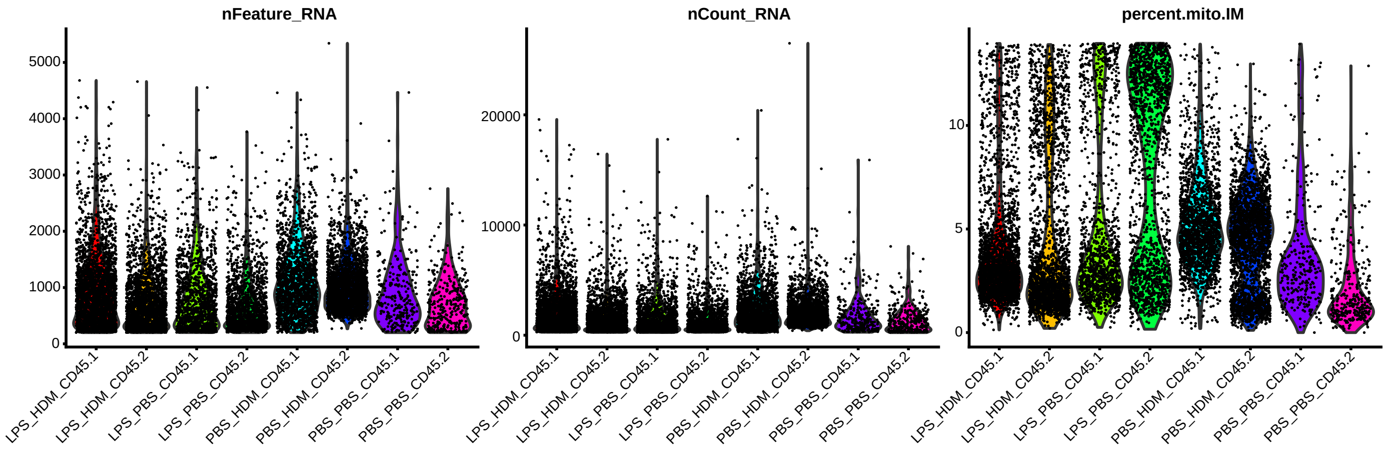

Supplemental Figure S2. *Increased mitochondrial gene cutoff from initial IM clustering to remove cell cluster artifacts of damaged cells. Interstitial macrophage clustering was assessed for expression of mitochondrial genes.* A) During initial clustering, evidence of high mitochondrial gene expression was appreciated in one of the clusters of interest. B) This was also considered by violin pilots based on the different cell sources (recruited versus resident-derived) and exposure to define an improved cutoff for mitochondrial genes.

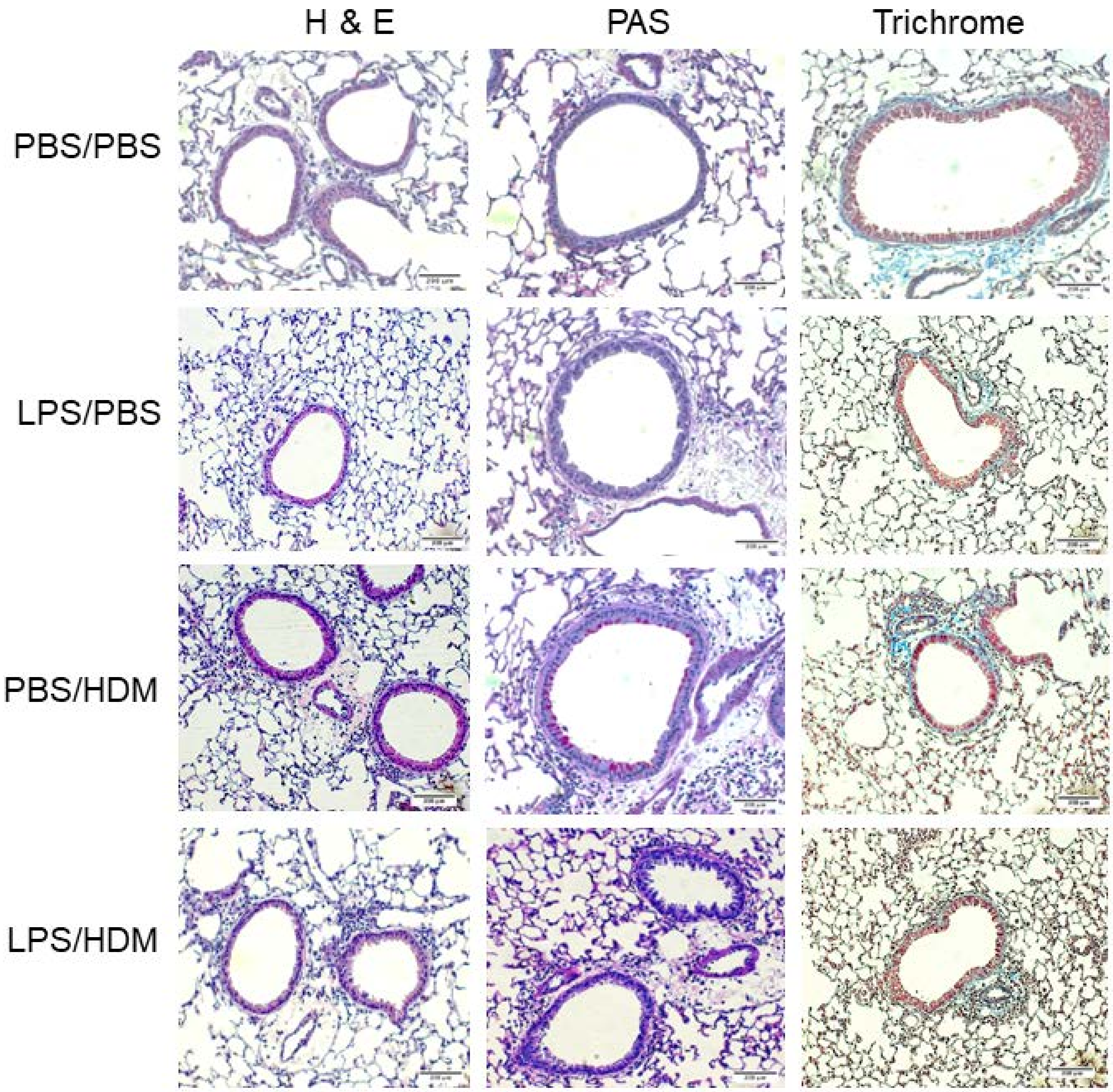

Supplemental Figure S3. *Representative images for H&E, Trichrome, and PAS staining from the PBS_PBS, LPS_PBS, PBS_HDM, and LPS_HDM groups.* The left lung at the time of necropsy was isolated and inflated with formalin to fix the lung tissue. The tissue was then processed and sectioned. Staining was performed for hematoxylin and eosin (H & E) to assess inflammation, Periodic acid–Schiff (PAS) to assess for mucins, and trichrome to assess for evidence of fibrosis. Images are representative of sections from each exposure condition with a focus on similar regions (larger airways) of the lung tissue. The scale bar represents 200μm.

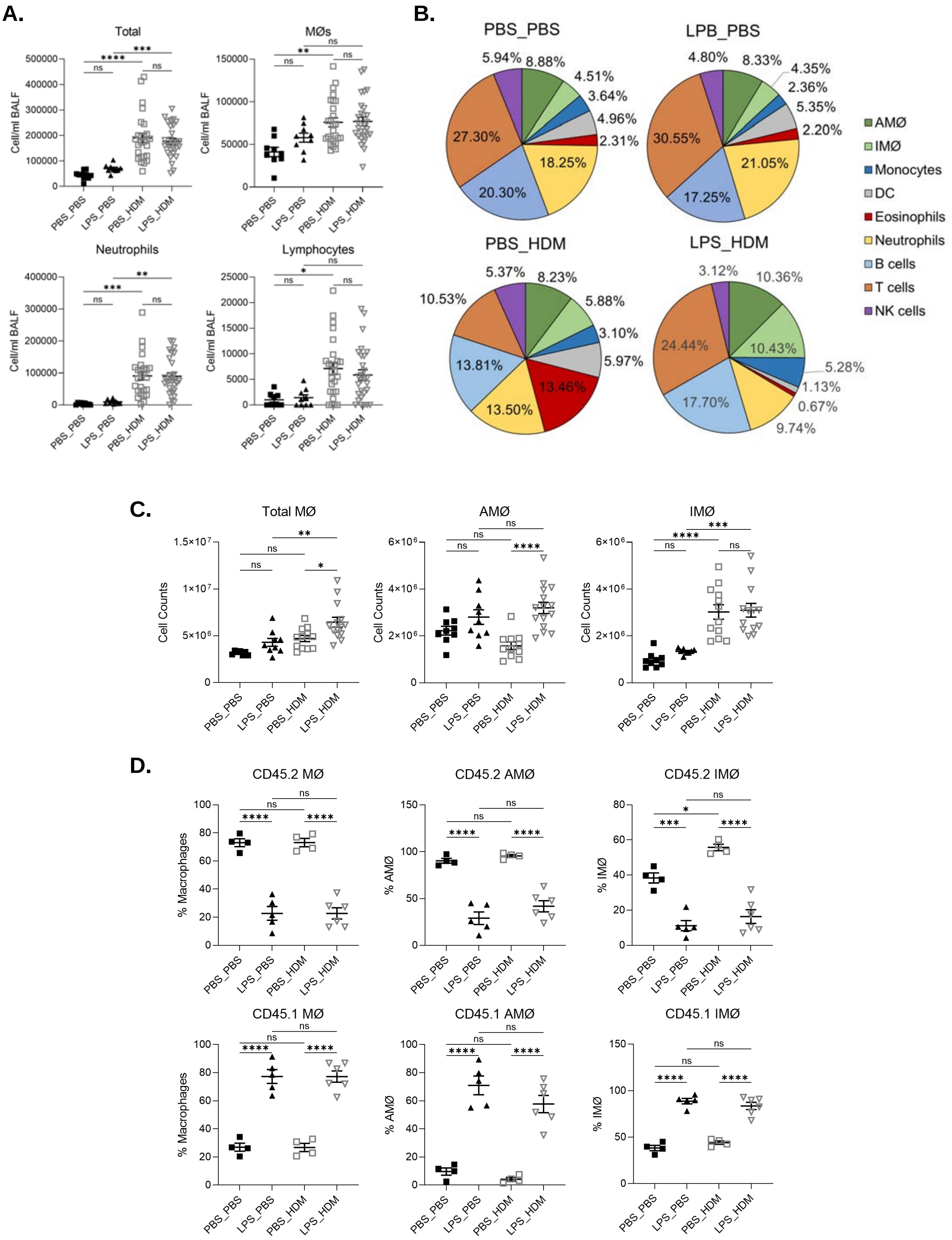
A.
Total
MØs
Neutrophils
Lymphocytes
Cell/ml BALF
PBS_PBS
LPS_PBS
PBS_HDM
LPS_HDM
B.
PBS_PBS
LPB_PBS
PBS_HDM
LPS_HDM
AMØ
IMØ
Monocytes
DC
Eosinophils
Neutrophils
B cells
T cells
NK cells
C.
Total MØ
AMØ
IMØ
Cell Counts
D.
CD45.2 MØ
CD45.2 AMØ
CD45.2 IMØ
CD45.1 MØ
CD45.1 AMØ
CD45.1 IMØ
% Macrophages
% AMØ
% IMØ

Supplemental Figure S4. *Characterization of BALF and lung tissue immune cell composition.* A) Total cell, macrophages, neutrophils, and lymphocyte counts in BALF following methacholine challenge in PBS and LPS pre-exposed animal, with and without subsequent HDM sensitization. B) Proportion of immune cells in the lung tissues of PBS and LPS pre-exposed animal, with and without subsequent HDM sensitization. The number depicted the mean of $N \geq 5$/group. C) Total macrophage, alveolar, and interstitial macrophage counts per lung in PBS and LPS pre-exposed animal, with and without subsequent HDM sensitization. D) Depicting proportion of CD45.2 vs. CD45.1 total macrophage, alveolar, and interstitial macrophages in PBS and LPS pre-exposed animal, with and without subsequent HDM sensitization. *$p<0.05$, **$p<0.005$, ***$p<0.0005$, ****$p<0.00005$ for other comparisons by 1-way ANOVA or Students T-test, n.s.=non-significant.

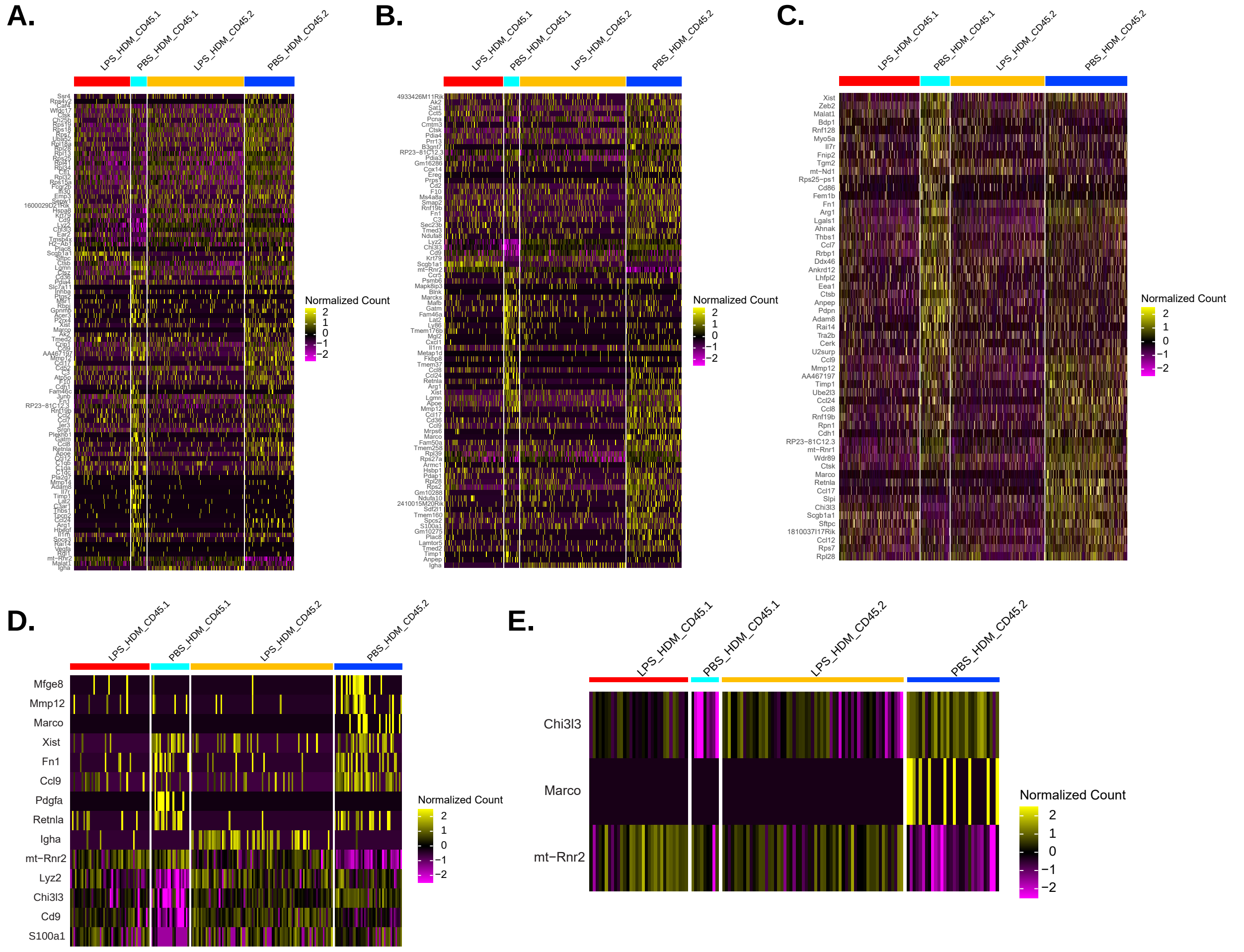

Supplemental Figure S5. *Heat map of gene expression for AMØ clusters.* Top differentially expressed genes were defined for each AMØ cluster and then were expressed as a heat map segregated based on the following conditions: LPS_HDM in CD45.1 cells (LPS_HDM_CD45.1, red bar), PBS_HDM in CD45.1 cells (PBS_HDM_CD45.1, light blue bar), LPS_HDM in CD45.2 cells (LPS_HDM_CD45.2, orange bar) and PBS_HDM in CD45.2 cells (PBS_HDM_CD45.2, blue bar). This allowed the comparison of gene expression patterns between exposure conditions based on cell origin in AM1 (A), AM2 (B), AM3 (C), AM4 (D), and AM5 (E) clusters.

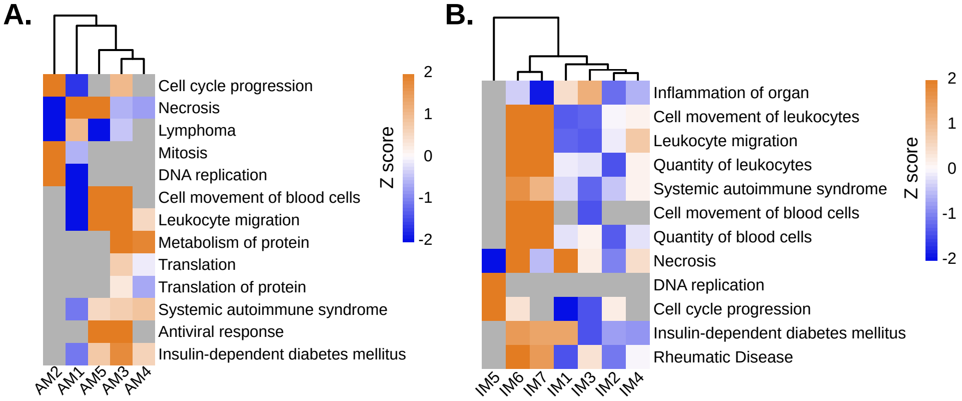

Supplemental Figure S6. *Pathway analysis per overall cluster in AMs and IMs, independent of exposure condition.* Ingenuity pathway analysis was performed on each alveolar macrophage (AM, A) and interstitial macrophage (IM, B) cluster independent of ontogeny or exposure condition to define overall patterns of the individual clusters. Hierarchical clustering was performed to define potential associations.

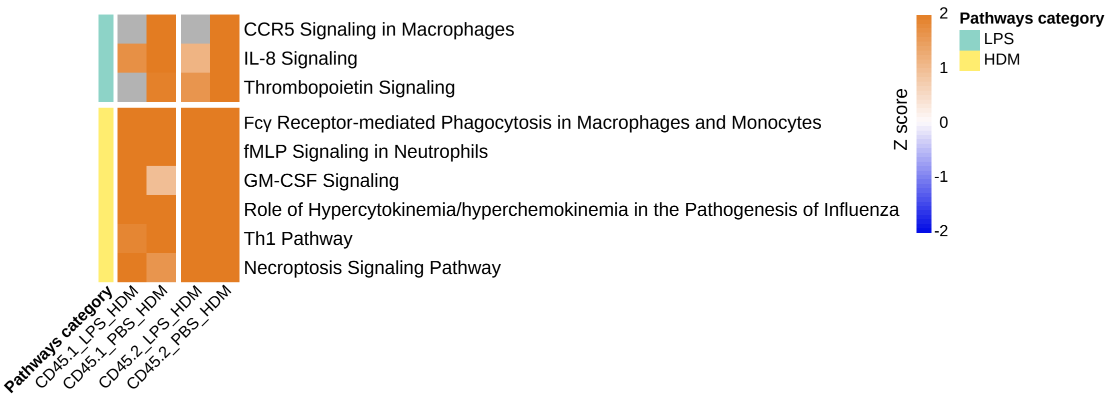

Supplemental Figure S7. *Pathway analysis in CD45.1 and .2 IM cluster 2 cells based on exposure conditions compared to PBS_PBS groups focused on common pathways in LPS and HDM responses across the exposure conditions.* The heatmap depicts pathways identified by IPA analysis (Z score >2 or <2) that are shared by LPS exposure or HDM sensitization.

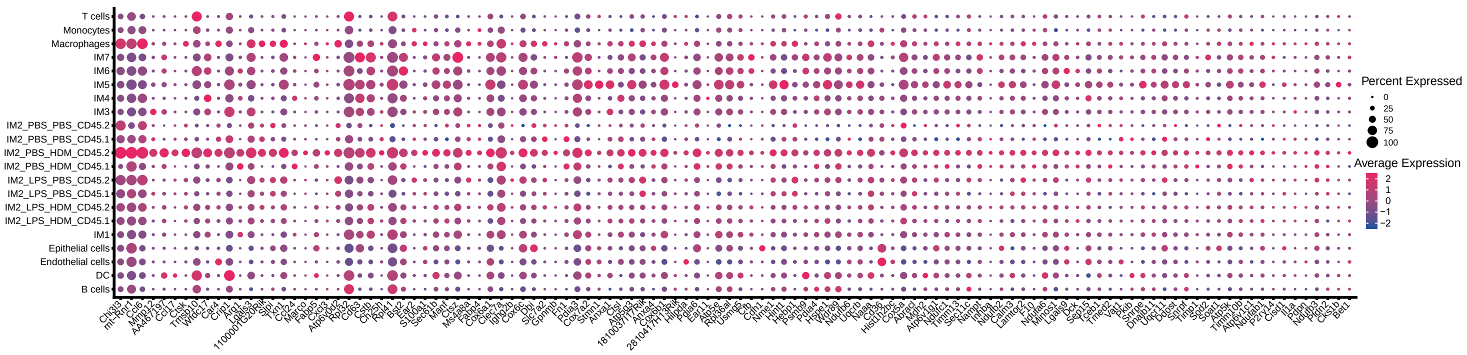

Supplemental Figure S8. *Gene analysis of all immune cells, interstitial macrophages, and the IM2 cluster based on exposure conditions defined specific markers for the IM2 cluster to use for examining cell tissue location.* Differential gene expression was performed to define potential markers that uniquely identified the IM2 cluster to consider for tissue immunofluorescence staining.

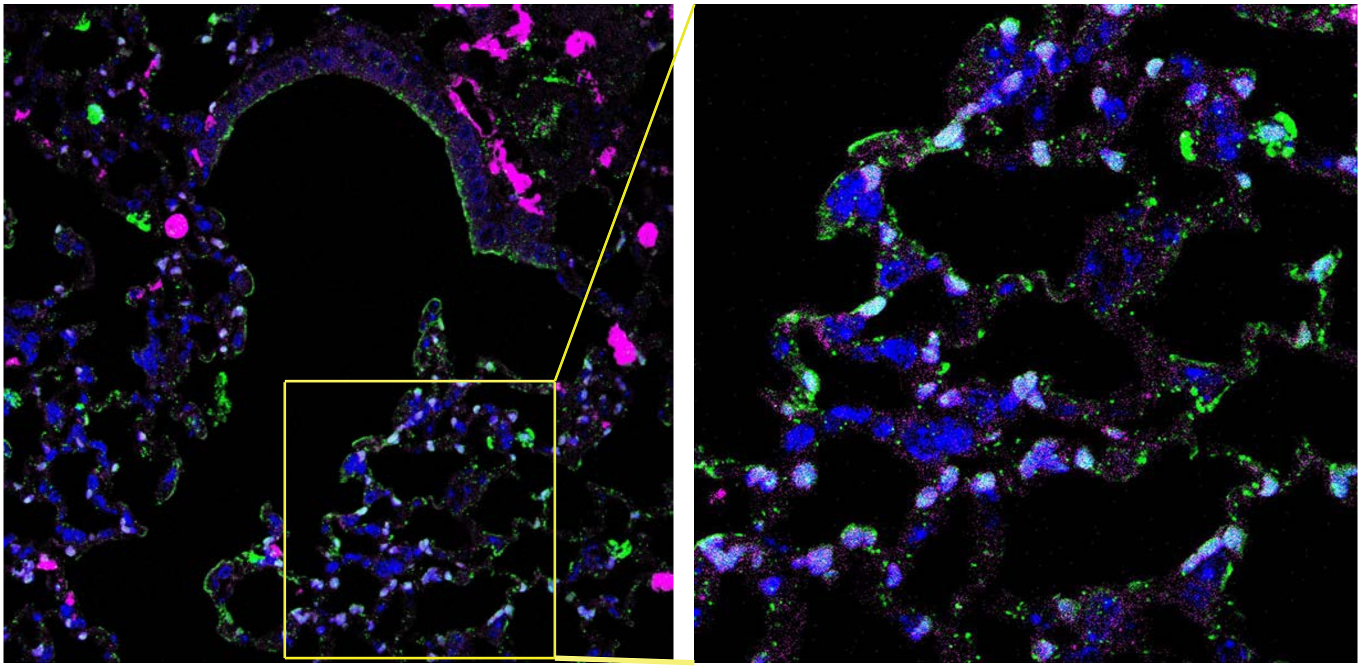

Figure S9. *Enlarged images of immunofluorescence staining for CD206 and Ctsk to define the location of the IM2 cluster in lung tissue. Image depicting the location of CD206+CTSK+ interstitial macrophages around the terminal bronchioles. CD206 (pink, pan-macrophage marker), DAPI (blue, to identify nuclei), and Ctsk (green, CD45.2 IM2 cluster). N = 3 animals* examined.
